# From molecular design to high performance: interpreting the combined effect of π-linkers and acceptor groups in benzotriazole-based non-fullerene acceptors

**DOI:** 10.1039/d6ra05880a

**Published:** 2026-10-19

**Authors:** Komal Ateeq, Muhammad Kamran Shehzad, Yasir Altaf, Maaz Ur Rehman, Rabia Bassran, Muhammad Farhan Waris, Fahim Ahmed, Farasat Haider, Basit Ali

**Affiliations:** a Department of Chemistry, Division of Science and Technology, University of Education Lahore 54770 Pakistan; b School of Materials Science and Engineering, Hefei University of Technology Hefei 230009 China; c Department of Physics, University of Sargodha 40100 Sargodha Pakistan; d International Graduate School of Nanoscience and Technology, College of Interdisciplinary and Integrative Studies, Chulalongkorn University Bangkok 10330 Thailand; e Department of Chemistry and Materials Science, School of Chemical Engineering, Aalto University P.O. Box 16100 FI-00076 Aalto Finland basit.ali@aalto.fi

## Abstract

The combined effect of π-linkers and terminal groups presents a powerful yet underexplored strategy for boosting the performance of benzotriazole (BZ)-based non-fullerene acceptors (NFAs). In this computational study, we employ density functional theory (DFT) and time-dependent DFT (TD-DFT) to design and evaluate the photovoltaic performance of five benzotriazole (BZ)-core-based non-fullerene small molecule acceptors (BD1–BD5). These molecules are engineered by modifying the terminal acceptor groups of a reference structure (BZE-31) and incorporating a methyl thieno[3,4-*b*]thiophene-2-carboxylate π-linker. The designed molecules exhibit a pronounced bathochromic shift in absorption (*λ*_max_ = 690–939 nm in chloroform) and significantly reduced optical band gaps (1.66–2.24 eV) compared to the reference. In particular, BD4, functionalized with a 4-methylene-5-oxocyclopent-1-ene-1-sulfonyl chloride end-group, demonstrates the most promising optoelectronic profile. It achieves the narrowest band gap (1.66 eV), the highest red-shifted absorption (939 nm), the lowest exciton binding energy (0.31 eV), and an exceptional short-circuit current density (*J*_sc_ ≈ 22 mA cm^−2^). This superior optoelectronic synergy transforms into an idealized theoretically estimated power conversion efficiency (PCE) of 23.48% for BD4. Concurrently, BD1 (with a malononitrile end-group) also shows superior performance, with a high predicted PCE. A comprehensive analysis of frontier molecular orbitals, density of states, transition density matrices, and global reactivity descriptors confirms effective intramolecular charge transfer and favorable charge separation in the designed systems. The estimated PCEs for the most promising candidates, BD4 and BD1, reach 23.48% and 22.61%, respectively. This work provides a detailed theoretical blueprint demonstrating that π-linker incorporation and strategic terminal group engineering work in a combined manner and are highly effective strategies for tailoring the optoelectronic properties of BZ-based NF-SMAs. The high-performance candidates (BD1 and BD4) offer valuable theoretical insights for the experimental synthesis of next-generation, high-efficiency organic photovoltaic materials.

## Abstract 2

This study introduces a π-linker of C_8_H_6_O_2_S_2_ in benzotriazole-core-based non-fullerene small-molecule acceptors and terminal group engineering, among which the –SO_2_Cl-functionalized BD4 is shown to have the lowest exciton binding energy of 0.34 eV.

## Graphical abstract

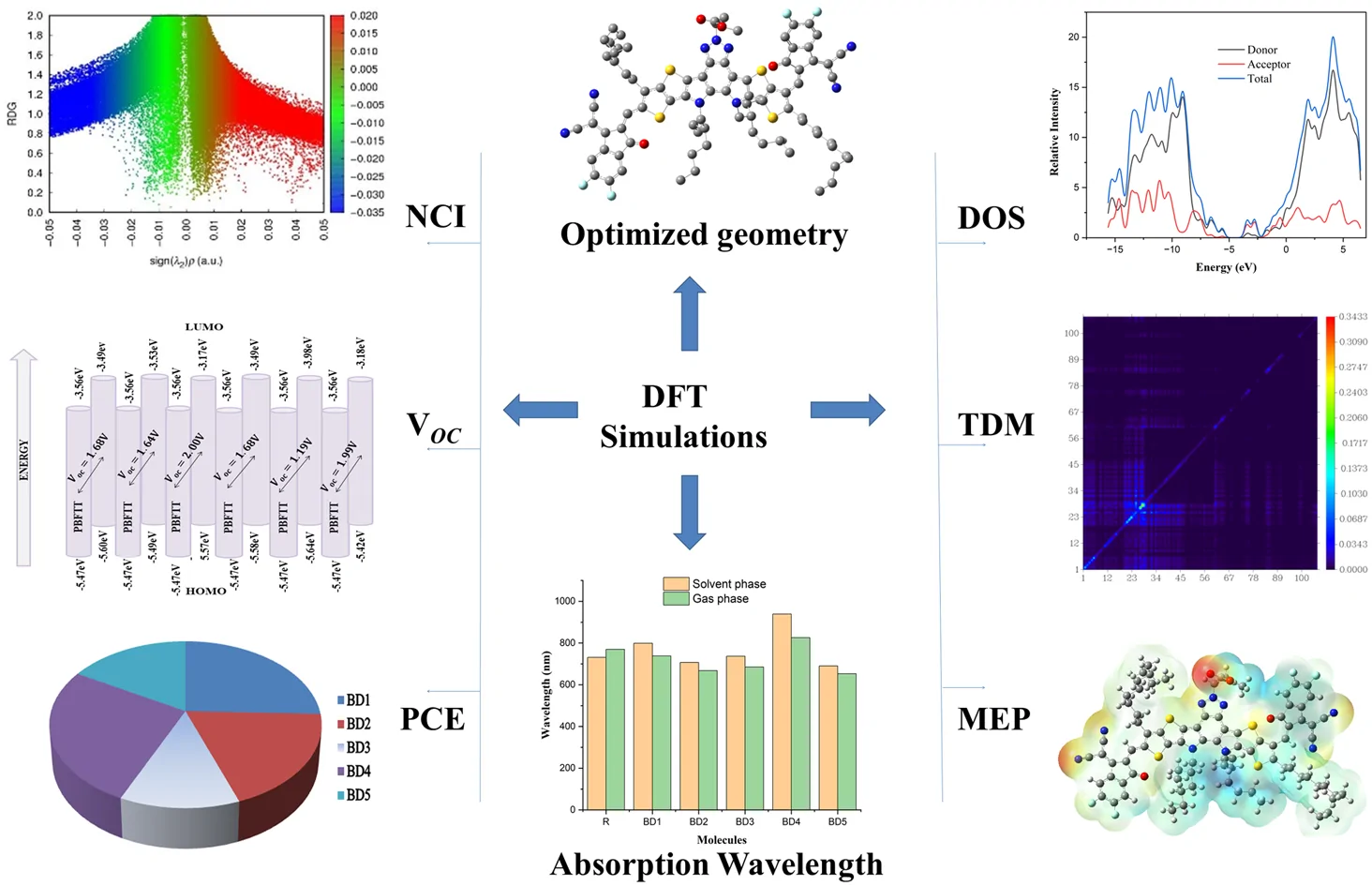

## 1 Introduction

Organic solar cells (OSCs) have emerged as a promising alternative due to their potential for low-cost, solution-processable fabrication, mechanical flexibility, lightweight nature, and compatibility with high-throughput roll-to-roll manufacturing.^1^ A landmark in this field is the emergence of Y6 and its derivatives; a class of A–DA′D–A type small-molecule acceptors (SMAs) that have propelled PCEs to new heights.^2^

Non-fullerene acceptors (NFAs) offer distinct advantages, such as versatile molecular structures, strong and tunable absorption profiles, and more straightforward synthesis.^3^ The pairing of a well-designed small-molecule NFA with a complementary polymer donor is now recognized as one of the most effective strategies for achieving high-performance organic solar cells.^2^ Enhancing the efficiency of non-fullerene organic solar cells requires simultaneously optimizing three key parameters: broadening the absorption spectrum, minimizing the optical band gap to promote charge generation, and reducing energy losses to achieve a high open-circuit voltage (*V*_oc_).^4^ Distinct structural modifications on small-molecule acceptors (SMAs) play specialized roles in tuning their optoelectronic and photovoltaic properties. Modifications to the central core primarily modulate electronic energy levels, absorption characteristics, and intermolecular packing.^5^ Altering the terminal acceptor (end) groups directly influences optical properties, charge transfer dynamics, and molecular stacking, which collectively enhance light harvesting and charge transport.^6^ Saghir *et al.* designed a series of molecules (M1–M6) using a thiazolothiazole-based NFA *via* a terminal group engineering approach. This strategy effectively tuned their optoelectronic properties and electronic structure.^7^ Extension in conjugation *via* π-linkers plays a significant role in the reduction of the energy gap and red-shift in absorption spectra.^8^

Quinoxaline and benzothiadiazole are widely used for central core modification due to their abundant reaction sites and subsequent molecular alteration.^9,10^ Despite the outstanding achievement of Y-series NFA central cores, further enhancement of device performance is still achievable. Benzotriazole offers enhanced solubility in organic solvents with a well-matched energy level and appropriate absorption. In 2022, Zhou *et al.* synthesized a series of benzotriazole-based NFAs by fluorination and side chain regulation. Remarkable light absorption and π–π stacking enhance the solar cell efficiency up to 10.3%.^11^ Meanwhile, terminal group engineering on benzotriazole-based NFAs emerges as an appropriate approach to increase device performance, as reported by Tahir *et al.*^12^ Although these significant approaches enhance the overall efficiency of organic solar cells, further improvement is still possible. Motivated by this research gap, we have utilized the combined effect of both terminal group and π-linker engineering to boost the PCE of studied molecules.

In 2024, Zhou *et al.* designed and synthesized a series of benzotriazole (BZ)-based small-molecule acceptors (SMAs) with varying N-substituted side chains on the BZ core.^13^ The side chains included *n*-butyl (C4), 2-ethoxy-2-oxoethyl (E22), 3-methoxy-3-oxopropyl (E31), and 6-ethoxy-6-oxohexyl (E62), leading to the molecules designated as BZ-C4, BZ-E22, BZ-E31, and BZ-E62, respectively. When blended with the polymer donor PM6, these ester-functionalized acceptors yielded organic solar cells (OSCs) with comparable open-circuit voltages (*V*_oc_). Among them, the device based on BZE-31 achieved the highest power conversion efficiency (PCE) of 18.33%, with a *V*_oc_ of 0.875 V and an outstanding fill factor (FF) of 76.7%. This promising experimental result establishes BZE-31 as an excellent candidate for further theoretical investigation. The present computational study is motivated by the goal of understanding the fundamental structure–property relationships in this system and proposing designs to enhance its photovoltaic performance beyond the reported benchmark.

Herein, five novel molecules (BD1–BD5) were designed using a dual modification approach where a π-spacer named methyl thieno[3,4-*b*]thiophene-2-carboxylate and five different terminal groups, as reported in the molecular design section 2.1, were inserted while preserving the central core of the reference molecule (R). The selection of terminal groups is based on their electron-withdrawing ability and structure compatibility with a BZ-based molecular framework. The molecules BD1 and BD4, having end groups such as 2-(2-methylene-3-oxo-2,3-dihydro-1*H*-ylidene)malanonitrile and 4-methylene-5-oxocyclopent-1-ene-1-sulfonyl chloride, exhibit higher *J*_SC_ and PCE, which may be the result of the combined influence of both the electron-withdrawing group and π-spacer. Overall, our strategy aims to evaluate how the simultaneous modulation affects the photovoltaic performance of organic solar cells, thereby offering new possibilities for the development of efficient acceptor materials for OSCs.

## 2 Computational details

Density functional theory (DFT) calculations, performed with the Gaussian 16 software package,^14^ were employed to investigate the photophysical properties of the designed molecular systems. The influence of the solvent environment (chloroform) was incorporated at both the ground-state (DFT) and excited-state (TD-DFT) levels of theory using the Integral Equation Formalism Polarizable Continuum Model (IEFPCM). In this study, the absorption profile of the reference molecule (R) was determined using TD-DFT.^15^ It involved using four different functionals, B3LYP,^16^ CAM-B3LYP,^17^ MPW1PW91,^18^ and ωB97XD,^19^ each with the 6-31G(d,p)^20^ and 6-311G(d,p) basis sets. As shown in Fig. 1, the obtained absorption maxima *via* these functionals are 797 nm, 532 nm, 731 nm and 512 nm, respectively. The long-range corrected functional such as CAM-B3LYP and ωB97XD exhibits the greatest deviation in absorption spectra from the experimentally calculated value (754 nm). Therefore, the MPW1PW91 functional, which yielded an absorption maximum (*λ*_max_) of 731 nm, which is in closest agreement with the experimental value of 754 nm for BZE-31, was selected. Furthermore, the MPW1PW91 functional is known to perform reliably for extended π-conjugated systems and is selected for all subsequent calculations on the designed molecules. First of all, the molecular geometries of all designed derivatives were optimized as represented in Fig. 2 to determine the most stable three-dimensional structure of the molecules. Following geometry optimization, key electronic properties of the molecules, including the energies of the highest occupied molecular orbital (HOMO), the lowest unoccupied molecular orbital (LUMO), and the exciton binding energy, were calculated. In addition, the density of states (DOS) for the optimized structures was computed and analyzed using PyMOlyze 1.1.^21^ A range of key electronic and photovoltaic properties was calculated, including exciton binding energy (*E*_b_),^22^ dipole moment (*µ*),^23^ and open-circuit voltage (*V*_oc_).^24^ Furthermore, to gain detailed insight into the nature of the electronic excitations, a transition density matrix (TDM) analysis^25^ was performed using the Multiwfn software.^26^

**Fig. 1:**
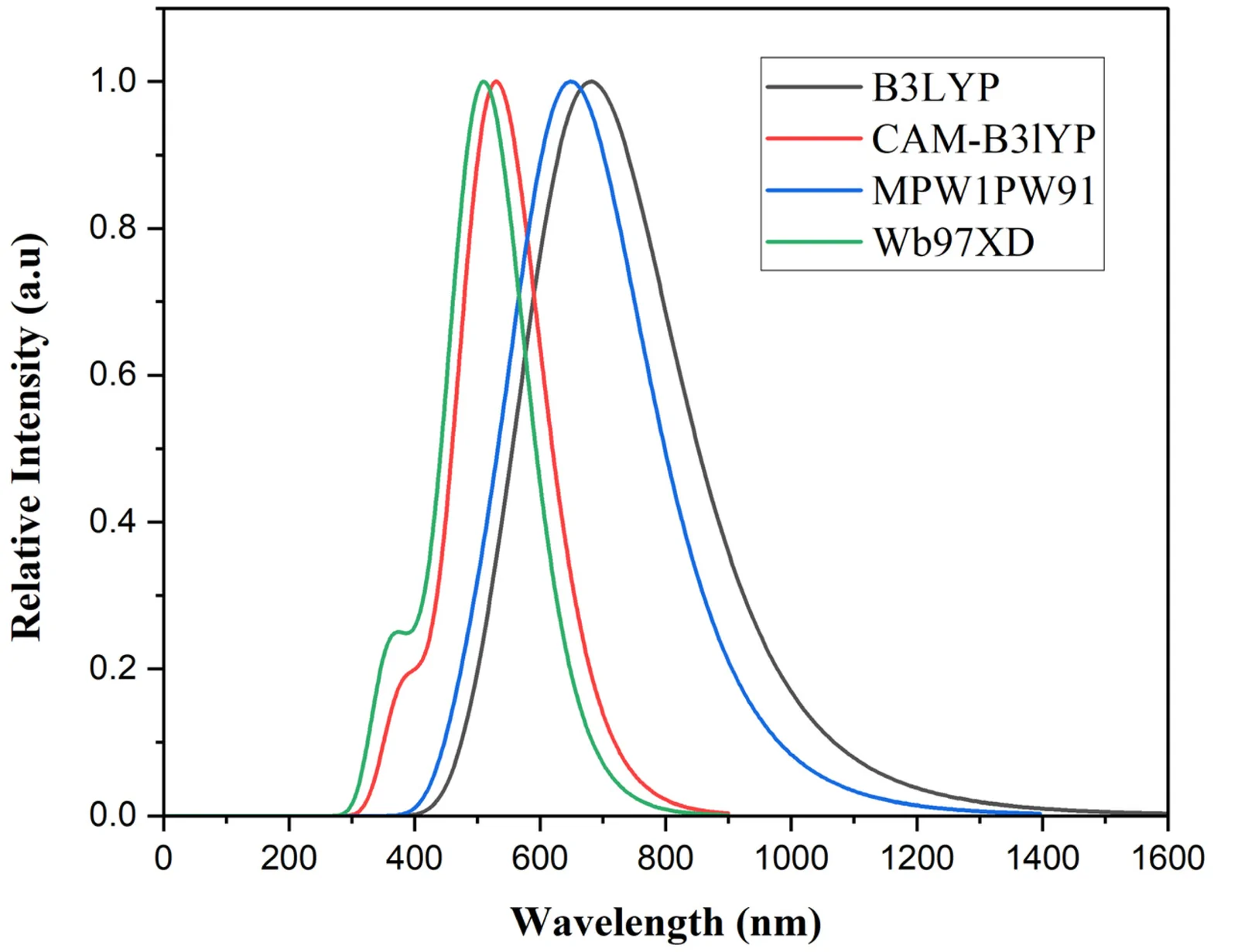
Reference benchmarking on different functionals.

**Fig. 2:**
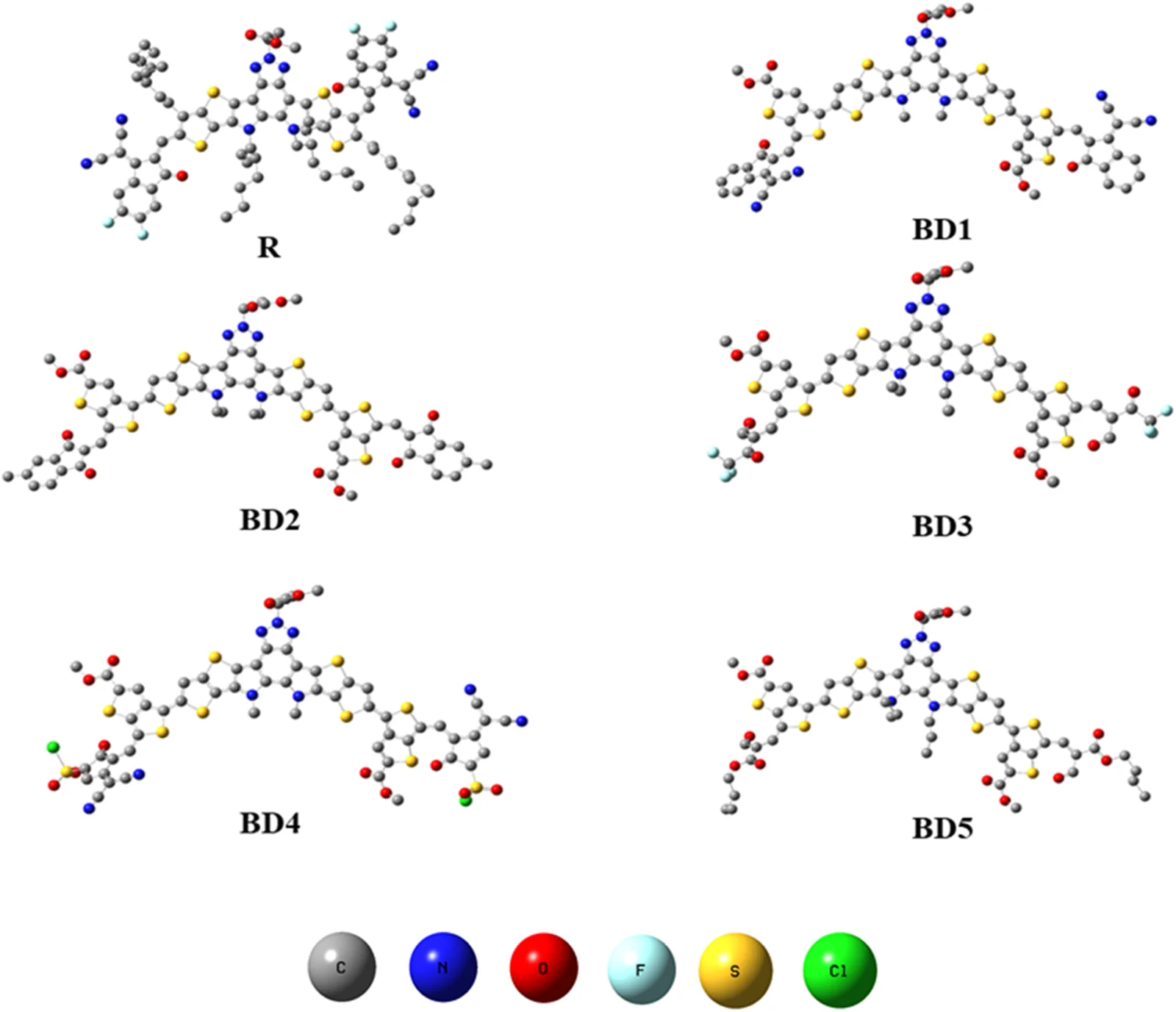
Optimized geometry of all designed molecules (BD1–BD5) excluding hydrogen.

### 2.1 Molecular design

New molecules are designed *via* a dual-modification approach by utilizing π-spacers and terminal groups. The selection of terminal groups is based on their electron-withdrawing ability, structural compatibility with a benzotriazole-based molecular framework, and extended conjugation. These distinctive features enable them to influence the optical absorption, electronic structure, intermolecular charge transfer, and photovoltaic properties of organic solar cells, ultimately enhancing the device performance. This design strategy helps assess the effect of various terminal groups on solar cells while maintaining the molecular backbone.

In the current study, the end-capped moieties of R were modified as illustrated in SI Fig. S2, such as 2-(2-methylene-3-oxo-2,3-dihydro-1*H*-ylidene)malononitrile (BD1), 5-methyl-2-methylene-1*H*-indene-1,3(2*H*)-dione (BD2), 4,4,4-trifluoro-2-methylene-3-oxobutanal (BD3), 4-methylene-5-oxocyclopent-1-ene-1-sulfonyl chloride (BD4), butyl 2-formylacrylate (BD5). To maintain computational feasibility while preserving the essential electronic structure, the *N*-alkyl side chain of the benzotriazole core was simplified to a methyl group (–CH_3_) in all molecules. Although alkyl chains influence the molecular packing dipole moment and conformational flexibility, they have a negligible effect on HOMO/LUMO energies, absorption maxima, and dipole moment. The simplification of alkyl chains ensures that differences in photovoltaic properties originate from the incorporation of the π-linker and various side groups, rather than from the alkyl chains themselves.

## 3 Result and discussion

### 3.1 UV-visible spectral characteristics

The optical properties of organic semiconductors are critical determinants of their photovoltaic performance. The absorption spectra of the reference (R) and designed molecules (BD1–BD5) were computed at the MPW1PW91/6-311G(d,p) level of theory. Key optical parameters, including maximum absorption wavelength (*λ*_max), oscillator strength (*f*), light-harvesting efficiency (LHE), dipole moment (*µ*), and excitation energy (*E*_X_), calculated for both the gas and solvent phases, are presented in Table 1 and SI Fig. S2.

**Table 1:** Calculated values of absorption profile (*λ*_max_), oscillator strength (*f*), Light Harvesting Efficiency (LHE), excitation energy (*E*_x_), molecular orbital (MO) assignment from HOMO–LUMO (H → L) exciton binding energy (*E*_b_), and in solvent (chloroform) phase

Molecules	*λ*_max_ (nm)	Experimental (nm)	*f*	LHE	*E*_x_ (eV)	Major MO assignment H → L	*E*_b_ (eV)
R	731	754	0.16	0.31	1.69	97%	1.98
BD1	799		2.71	0.998	1.55	94%	0.41
BD2	707		2.76	0.998	1.75	94%	1.75
BD3	737		2.52	0.996	1.68	97%	0.72
BD4	939		1.23	0.942	1.32	91%	0.34
BD5	690		2.47	0.996	1.79	95%	0.45

All newly designed molecules exhibit a significant increase in *λ*_max_ compared to the reference. This bathochromic (red) shift is a direct consequence of the strong electron-withdrawing character of the new terminal acceptor groups. These groups effectively extend the molecular conjugation, stabilize the LUMO energy level, and thereby reduce the optical band gap (*E*_g_). Consequently, there is a clear negative correlation between the optical band gap and the *λ*_max_ value. The enhanced absorption profiles and lower band gaps collectively contribute to an improved theoretical light-harvesting capacity of the designed molecules.

Among the designed molecules, BD4 exhibits the most pronounced bathochromic (red) shift, with a maximum absorption (*λ*_max_) at 939 nm in chloroform, which lies within the near-infrared (NIR) region. This is attributed to the introduction of the strong electron-withdrawing sulfonyl chloride (–SO_2_Cl) end group. The overall absorption trend follows the order: BD4 (939 nm) > BD1 (799 nm) > BD3 (737 nm) > BD2 (707 nm) > BD5 (690 nm). This trend is directly correlated with the strength and electronic nature of the terminal acceptor groups, which enhance donor–acceptor interactions, promote intramolecular charge transfer, lower the excitation energy, and shift absorption to longer wavelengths.

Notably, both BD4 (*λ*_max_ = 939 nm) and BD1 (*λ*_max_ = 799 nm) demonstrate absorption maxima near 800 nm or exceeding 800 nm, positioning them as promising candidates for NIR-selective photodetection or as NIR-absorbing components in tandem or semi-transparent organic solar cells. The extension of absorption into the NIR region is crucial for harvesting a broader portion of the solar spectrum, particularly the underutilized infrared photons, which can potentially lead to higher photocurrent generation.

BD4 demonstrates superior NIR absorption due to the distinctive electronic effect of its –SO_2_Cl group, which promotes extensive conjugation, high molecular planarity, and a strong π–π* electronic transition. In contrast, the malononitrile (–C(CN)_2_) acceptor in BD1, while effective, can sometimes lead to a less planar molecular geometry and involves weaker n–π* transitions, resulting in absorption at a shorter wavelength, albeit still within the NIR window. For BD3, the 4,4,4-trifluoro-2-methylene-3-oxobutanal end group contains highly electronegative fluorine atoms. This electronegativity can localize π-electron density, potentially disrupting optimal conjugation and reducing absorption intensity in the visible-to-near-infrared region compared to the more resonance-capable –CN groups in BD1.

All molecules exhibit enhanced absorption in the solvent (chloroform) phase compared to the gas phase, due to solvatochromic effects as represented in S2, SI Table 1 and S1. The gas-phase absorption trend mirrors that in solution: BD4 (826 nm) > BD1 (738 nm) > BD3 (685 nm) > BD2 (668 nm) > BD5 (653 nm). In both phases, BD4 displays a remarkable red shift relative to the reference molecule (R), indicating its superior potential for light harvesting, particularly in the NIR spectrum. These values are detailed in SI Table S1, and a visual comparison is provided in Fig. 4.

The introduction of strong electron-accepting groups effectively lowers the energy of the lowest unoccupied molecular orbital (LUMO), thereby reducing the HOMO–LUMO gap (band gap, *E*_g_) and enhancing absorption. The primary electronic transition in these systems is characterized by a HOMO → LUMO excitation. The calculated contribution of this transition to the first excited state is 94%, 94%, 97%, 91%, and 95% for BD1 through BD5, respectively, in the solvent phase. Such dominant HOMO → LUMO character signifies efficient intramolecular charge transfer, which is a key indicator of favorable exciton dissociation and a high theoretical open-circuit voltage (*V*_oc_). This computational study successfully maps the structure–property relationships, indicating the combined role of π-linker and end-group engineering in tailoring optoelectronic properties to improve the theoretical performance of organic solar cells.

### 3.2 Frontier molecular orbitals (FMOs)

The distribution of charge and the energy levels of frontier molecular orbitals, specifically the highest occupied molecular orbital (HOMO) and the lowest unoccupied molecular orbital (LUMO), are fundamental descriptors of a molecule's electronic structure. The alignment of the HOMO and LUMO energy levels is crucial for efficient charge transfer, governing processes such as exciton dissociation and electron–hole transport in organic solar cells (OSCs).^27^ In the context of OSCs, FMO analysis provides direct insight into photoexcitation, light absorption, and the efficiency of charge transfer (CT). It is also essential for calculating key photovoltaic parameters, including the open-circuit voltage (*V*_oc_) and exciton binding energy (*E*_b_). The HOMO and LUMO energies for the designed molecules were calculated at the DFT/MPW1PW91/6-311G(d,p) level of theory and listed in Table 2, with the corresponding FMO surfaces depicted in Fig. 3. The resulting FMO surfaces, along with the corresponding HOMO (*E*_HOMO_) and LUMO (*E*_LUMO_) energy levels and band gaps (*E*_g_), are presented in Fig. 5 and S3. The computed *E*_HOMO_ values for the tailored molecules BD1–BD5 are −5.49, −5.57, −5.58, −5.64, and −5.42 eV, respectively. The corresponding *E*_LUMO_ values are −3.53, −3.17, −3.49, −3.98, and −3.18 eV. From these values, the HOMO–LUMO gaps (*E*_g_) are calculated to be 1.96, 2.20, 2.09, 1.67, and 2.24 eV for BD1 to BD5, respectively. A graphical representation of the HOMO and LUMO energy levels and the derived band gaps is provided in SI Fig. S3. The FMO analysis reveals how modifications to the terminal acceptor groups directly influence the photovoltaic properties. Changes in the HOMO and LUMO energies (effectively the valence and conduction band edges) alter the fundamental band gap and the driving force for charge transfer, thereby impacting the material's optoelectronic conductivity and device performance.

**Table 2:** HOMO–LUMO energy and bandgap of R and the designed molecule (BD1–BD5)

Molecules	HOMO (eV)	LUMO (eV)	Bandgap (eV)
R	−5.60	−3.49	2.11
BD1	−5.49	−3.53	1.96
BD2	−5.57	−3.17	2.20
BD3	−5.58	−3.49	2.09
BD4	−5.64	−3.98	1.66
BD5	−5.42	−3.18	2.24

**Fig. 3:**
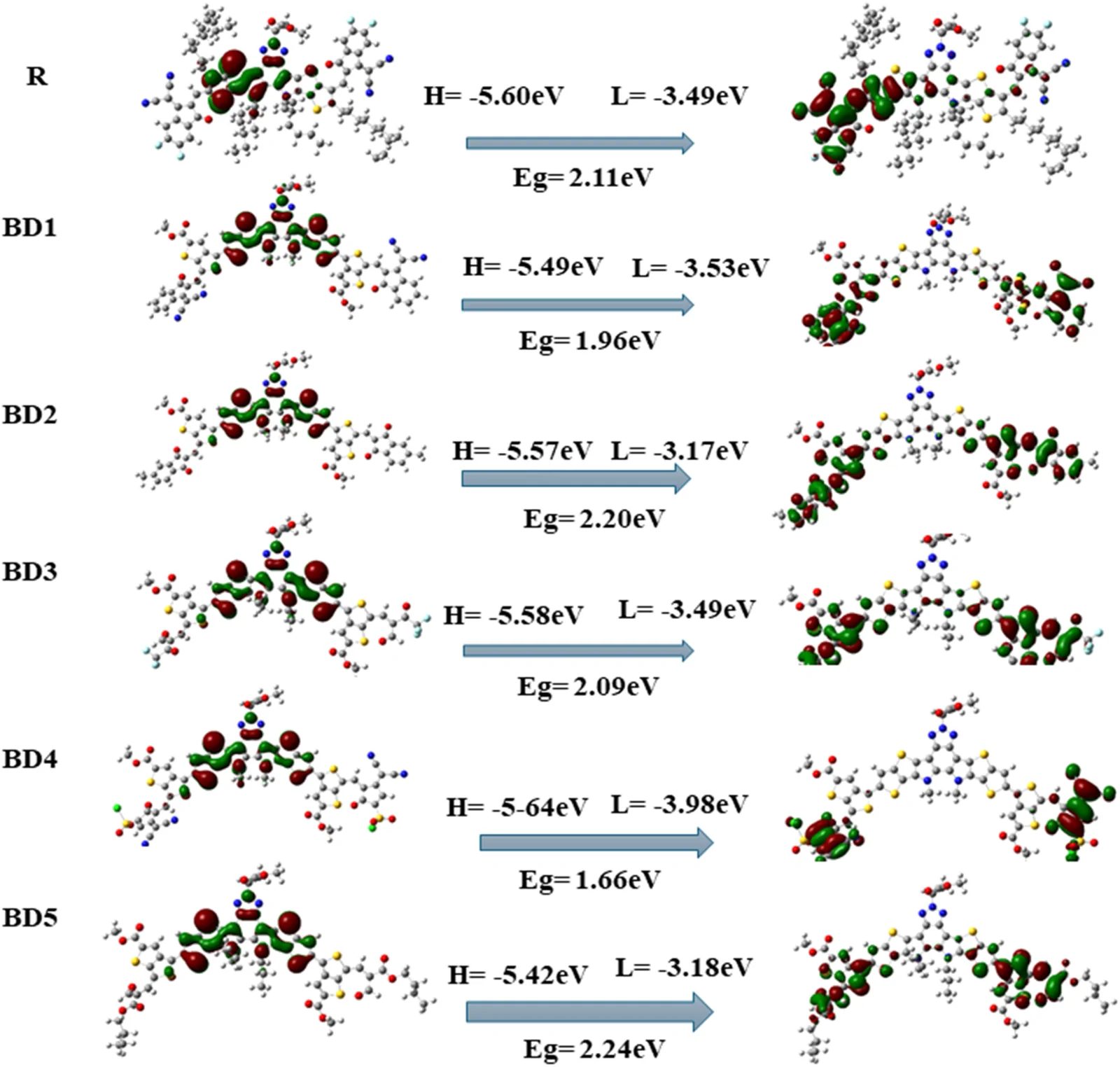
FMO diagrams of R and designed molecules (BD1–BD5).

The HOMO–LUMO band gap (*E*_g_) of the reference molecule (R) is 2.11 eV. The introduction of strong electron-withdrawing terminal groups in the designed derivatives systematically modulates this value. This variation is directly attributable to the specific electronic nature of the end-capped acceptor moieties: the dicyanomethylene (–C(CN)_2_) group in BD1, the 5-methyl-2-methylene-1*H*-indene-1,3(2*H*)-dione in BD2, the 4,4,4-trifluoro-2-methylene-3-oxobutanal group in BD3, the 4-methylene-5-oxocyclopent-1-ene-1-sulfonyl chloride (–SO_2_Cl) group in BD4, and the butyl 2-formylacrylate group in BD5. Among all molecules, BD4 exhibits the narrowest band gap (1.67 eV), which is primarily due to the strong electron-withdrawing and conjugation-extending effect of its sulfonyl chloride (–SO_2_Cl) acceptor. In general, the electron-withdrawing groups in BD1–BD5 effectively stabilize the LUMO energy level. This LUMO stabilization, relative to a less perturbed HOMO, is the key factor that reduces the HOMO–LUMO gap. In non-fullerene acceptors (NFAs), such targeted LUMO stabilization is a primary strategy for optimizing optoelectronic properties. A smaller band gap has two major beneficial effects for photovoltaic performance: it extends the absorption profile to longer wavelengths, enhancing light-harvesting and photocurrent generation. It modifies the interfacial band structure with the donor material, creating a stronger built-in electric field that facilitates charge separation upon photoexcitation. The efficient spatial separation of HOMO and LUMO densities, as seen in these systems, promotes intramolecular charge transfer, which is critical for achieving high solar cell efficiency. The bandgap is calculated by eqn (1).

1 *E*_g_ = *E*_HOMO_ − *E*_LUMO_

The calculated HOMO (*E*_HOMO_) and LUMO (*E*_LUMO_) energy levels determine the molecular band gap. BD5 and BD2 exhibit slightly larger band gaps than the reference (R), attributable to the relatively weaker electron-accepting character of their end groups. This result highlights the necessity of a strong acceptor for effective band gap reduction. The complete set of HOMO/LUMO energies is listed in Table 2, with the corresponding FMO surfaces depicted in Fig. 5.

### 3.3 Oscillator strength and excitation energy

The oscillator strength (*f*) quantifies the probability of an electronic transition, while the corresponding energy required is the excitation energy (*E*_X_). A high oscillator strength is predictive of efficient charge transfer, characterized by a low excitation energy, a broad absorption band, and a large molar extinction coefficient (*ε*).^28^ In photovoltaic materials, a high molar extinction coefficient is essential for effective light harvesting. The oscillator strength (*f*) for the first excited state (S_1_), which predominantly involves a HOMO → LUMO transition, was calculated for all molecules. A visual representation is provided in SI Fig. S4, and the complete data are tabulated in Table 1 and S1 SI. All designed derivatives exhibit a significant increase in oscillator strength compared to the reference molecule (R), which has values of *f* = 0.16 in chloroform and *f* = 0.05 in the gas phase. The highest value is observed for BD2. The order of oscillator strength in chloroform is: BD2 (2.76) > BD1 (2.71) > BD3 (2.52) > BD5 (2.47) > BD4 (1.23) > R (0.16). A similar trend holds in the gas phase: BD2 (2.53) > BD1 (2.49) > BD3 (2.24) > BD5 (2.22) > BD4 (1.22) > R (0.05). The higher oscillator strength values in the designed molecules indicate a significantly enhanced probability of interaction with incident photons, directly leading to superior light absorption. In non-fullerene acceptors, this stronger absorption translates to more efficient exciton generation. Following dissociation, these excitons contribute to a higher short-circuit current density (*J*_SC_). Consequently, the designed structures possess a high theoretical light-harvesting efficiency (LHE), a property that is fundamental for effective photoexcitation and performance in organic solar cells.

The excitation energy (*E*_X_) represents the energy required for the primary electronic transition, typically from HOMO to LUMO. A lower *E*_X_ value facilitates a more efficient intermolecular charge transfer (ICT) by easing the transition from the ground state to the excited state.^29^ Consequently, *E*_X_ and charge transfer efficiency exhibit an inverse relationship: a reduced *E*_X_ signifies enhanced ICT capability. A visual elucidation of the excitation process is provided in SI Fig. S5. The computed *E*_X_ for the reference molecule (R) is 1.69 eV in the solvent phase and 1.61 eV in the gas phase. All designed derivatives exhibit lower *E*_X_ values than R, indicating improved charge transfer characteristics. In solvent (chloroform), the designed molecules exhibit *E*_X_ as: BD1 (1.55 eV), BD2 (1.75 eV), BD3 (1.68 eV), BD4 (1.32 eV), BD5 (1.79 eV), while in the gas phase, these values are noted as: BD1 (1.67 eV), BD2 (1.85 eV), BD3 (1.81 eV), BD4 (1.49 eV), BD5 (1.89 eV). The reduction in *E*_X_, noticeable in BD4, is attributed to the introduction of strong electron-withdrawing terminal groups (–SO_2_Cl in BD4, –CF_3_ in BD3, –C(CN)_2_ in BD1). These groups stabilize the LUMO, effectively decreasing the energy gap between the π (HOMO) and π* (LUMO) orbitals. This smaller orbital energy separation facilitates the electronic transition, enhances the π–π* character of the excitation, and promotes efficient intramolecular charge transfer. The systematically lowered excitation energies confirm that all designed molecules are promising photoactive materials. The particularly low *E*_X_ of BD4, coupled with its narrow band gap, suggests it is the most effective candidate for enhancing light absorption and charge generation, thereby potentially boosting the overall performance of organic solar cells (OSCs).

### 3.4 Molecular electrostatic potential

Molecular electrostatic potential (MEP) maps help in predicting a three-dimensional visualization of the charge distribution in a molecule, identifying electrophilic (electron-deficient) and nucleophilic (electron-rich) regions critical for understanding charge separation and optoelectronic properties in organic solar cells.^30^ The MEP surfaces for the reference and all designed molecules (BD1–BD5) were calculated at the DFT/MPW1PW91/6-311G(d,p) level of theory. The resulting MEP surfaces are visualized in Fig. 4, where the electrostatic potential is represented by a color gradient. In the standard convention: red and yellow regions represent a negative electrostatic potential, indicating electron-rich (nucleophilic) sites. Blue regions represent a positive electrostatic potential, indicating electron-deficient (electrophilic) sites. Green regions denote a neutral or near-zero electrostatic potential. The potential typically increases in the order: red (most negative) < yellow < green < cyan < blue (most positive).^30^ Analysis of the MEP maps reveals distinct charge distribution patterns. As expected, intense red regions are localized on highly electronegative atoms such as oxygen and fluorine in the terminal acceptor groups, signifying a high electron density. In contrast, a faint blue or green color dominates the central hydrocarbon core, indicating a relative lack of charge. This clear color contrast can visualizes effective intramolecular charge separation between the electron-rich terminals and the electron-deficient core, a favorable feature for charge transfer upon photoexcitation. All modified molecules exhibit a pronounced red region at the terminal acceptor, confirming the presence of electron-withdrawing groups (EWGs). The intensity and extent of this red region vary, with BD3, BD1, and BD2 showing the most pronounced effects, which may be attributable to their strong –CF_3_, –C(CN)_2_, and carbonyl (–C=O) groups, respectively. The MEP analysis can support our approach that the designed molecules possess a favorable charge distribution with well-defined electron-rich (acceptor) and electron-deficient (donor) regions. This characteristic is necessary for efficient charge separation and transport, indicating their strong potential for improving the efficiency of OSC devices.

**Fig. 4:**
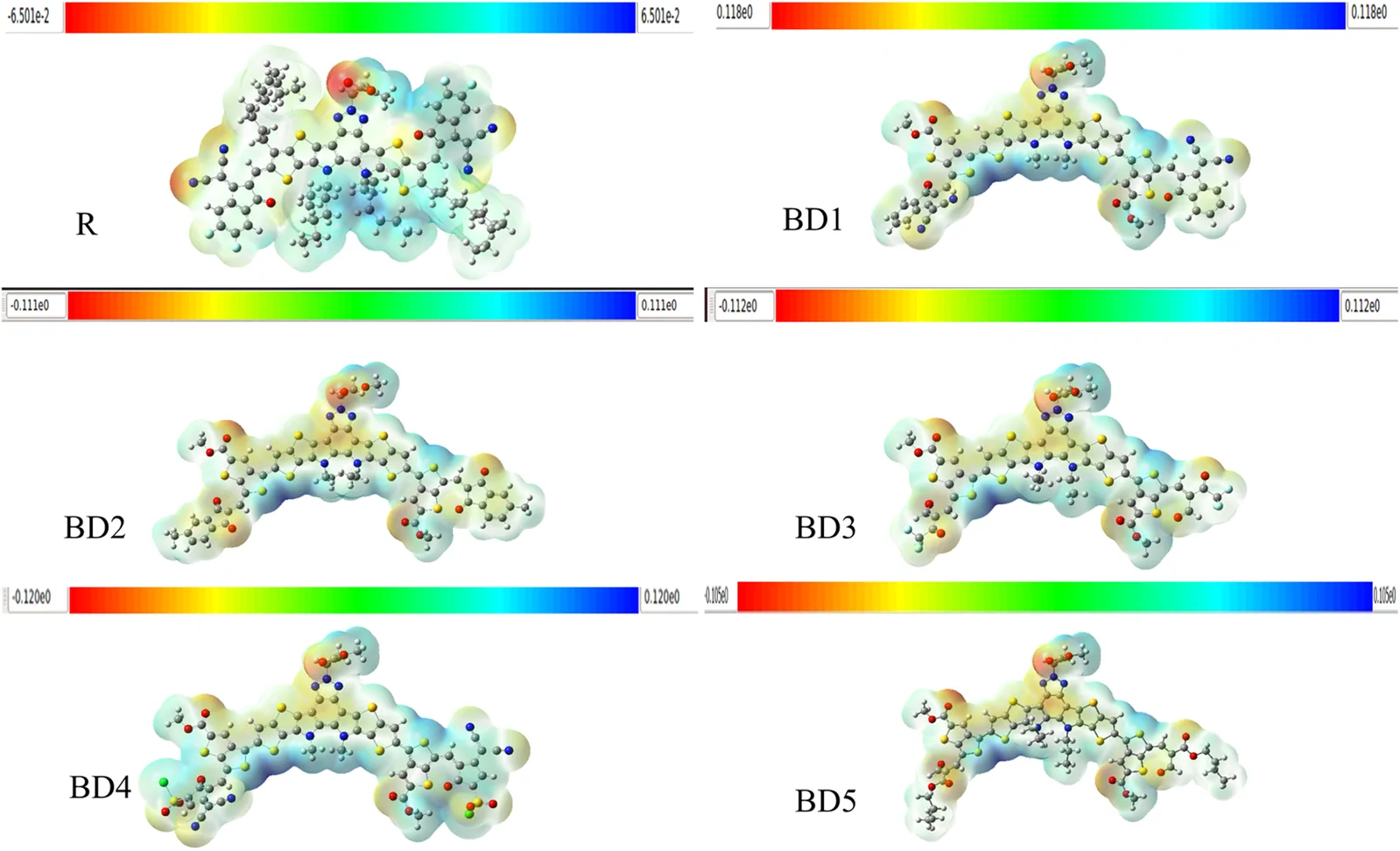
Visual representation of MEP plots for the designed molecules and R.

### 3.5 Density of states (DOS)

The results of the frontier molecular orbitals (FMOs) analysis were validated by calculating the density of states (DOS) for all designed molecules. The DOS provides detailed insight into the contribution of each molecular fragment; the donor core, the π-spacer, and the terminal acceptor groups to the electronic structure, absorption profile, and charge-transfer characteristics.^31^ The calculations were performed using density functional theory (DFT), and orbital-projected DOS was obtained with PyMOLyze 1.1. In the resulting plots (Fig. 5), contributions are color-coded: red (donor), green (π-spacer), and black (acceptor). The DOS spectra show distinct peaks corresponding to the HOMO (left side) and LUMO (right side) energy regions. The central region between these peaks represents the HOMO–LUMO energy gap. The plot clearly demonstrates how the donor, spacer, and acceptor fragments contribute to the frontier orbitals. A key observation is that the strong electron-withdrawing terminal groups significantly enhance the LUMO contribution localized on the acceptor moieties. This is evident as an intense LUMO peak on the right side of the spectrum, particularly notable in BD1, BD3, and BD5 compared to the reference molecule (R). The spatial segregation of DOS peaks associated with different molecular fragments indicates effective separation of electron density, a feature conducive to efficient intramolecular charge transfer. Such facilitated charge transport is crucial for improving charge generation and overall photovoltaic performance in organic solar cells.

**Fig. 5:**
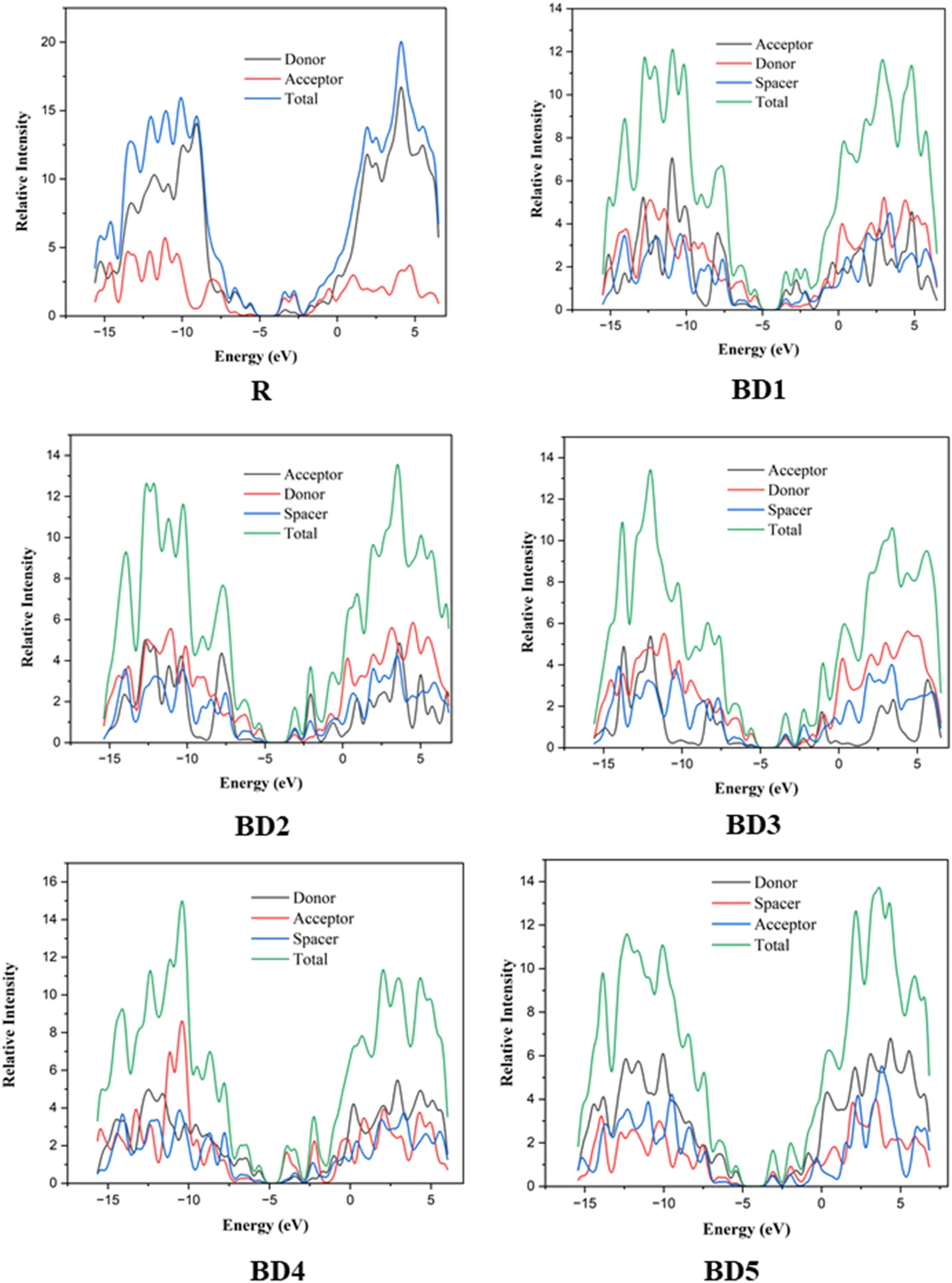
Illustration of DOS for R and BD1–BD5.

### 3.6 Exciton binding energy (*E*_b_)

The minimum energy required to separate a bound electron–hole pair (an exciton) into free charge carriers is known as the exciton binding energy (*E*_b_). This parameter is critical for determining the photovoltaic performance and efficiency of organic solar cells (OSCs). A lower *E*_b_ facilitates exciton dissociation, as it reduces the coulombic attraction that holds the electron and hole together. This weakened attraction allows the photogenerated charges to separate more readily and migrate towards their respective electrodes, thereby enhancing the device's current output.^32^ The short-circuit current (*J*_sc_) and *E*_b_ display an inverse relation as *E*_b_ successively increases the short-circuit current. For the calculation of *E*_b,_ the following eqn (2) is used.^33^

2 *E*_b_ = *E*_g_ − *E*_x_

Here, *E*_x_ and *E*_g_ are the excitation energy and bandgap, respectively. The difference between the energy gap and the first singlet excitation energy is used to calculate exciton binding energy. This is the most commonly used approximation for computational evaluation of exciton dissociation at the molecular level. However, these estimated values of exciton binding energy are obtained for an isolated molecule and do not deal with solid-state dielectric screening. Table 3 summarizes the computed exciton binding energies in the solvent phase. A comparison with the gas-phase values, which are listed in SI Table S1, allows for an analysis of the solvation effect on this parameter.

**Table 3:** Calculated values of *E*_g_ (bandgap), *E*_x_ (excitation energy), and *E*_b_ (exciton binding energy) through MPW1PW91 and basis set 6-311G (d,p)

Molecules	*E*_g_ (eV)	*E*_x_ (eV)	*E*_b_ = *E*_g_ − *E*_x_ (eV)
R	2.11	1.69	0.42
BD1	1.96	1.55	0.41
BD2	2.40	1.75	0.45
BD3	2.09	1.68	0.41
BD4	1.66	1.32	0.34
BD5	2.24	1.79	0.45

The computed exciton binding energies (*E*_b_) for the designed molecules in the solvent phase are significantly lower than that of the reference (R, 0.42 eV), as shown in Fig. S6. The values are: BD1 (0.41 eV), BD2 (0.45 eV), BD3 (0.41 eV), BD4 (0.34 eV), and BD5 (0.45 eV). A similar trend is observed in the gas phase, with calculated *E*_b_ values of: BD1 (0.33 eV), BD2 (0.36 eV), BD3 (0.32 eV), BD4 (0.31 eV), BD5 (0.35 eV), and R (0.40 eV). These data confirm that all tailored molecules exhibit reduced exciton binding relative to R, with BD4 consistently showing the lowest value in both phases. A graphical representation for the solvent phase is provided in Fig. 8. The low *E*_b_ of BD4 indicates that its photogenerated excitons are only weakly bound. This facilitates their dissociation into free charge carriers (electrons and holes), which can then be transported efficiently to the electrodes. This property is a direct consequence of the strong electron-accepting character of its terminal sulfonyl chloride (–SO_2_Cl) group, which enhances intramolecular charge transfer. Efficient exciton dissociation is a fundamental requirement for high short-circuit current and overall power conversion efficiency in organic solar cells. In contrast, BD2 exhibits the highest *E*_b_ among the designed series, implying more tightly bound excitons that are less prone to dissociation, which can limit device performance. Therefore, based on its lower exciton binding energy, BD4 emerges as the most promising candidate among the modified molecules, offering superior potential for enhanced light absorption and charge transport in photovoltaic applications.

### 3.7 Transition density matrix (TDM)

In photovoltaic research, the transition density matrix (TDM) is employed to map the electron density change during the S_0_ → S_1_ excitation, a key parameter for evaluating and engineering materials for solar cell applications.^30^ The transition density matrix (TDM) provides detailed insight into the geometry of the excited state, the specific roles of donor and acceptor moieties, and the spatial extent of electron delocalization upon photoexcitation for isolated molecules. TDM analysis was performed for both the reference (R) and the designed molecules to quantify the magnitude and direction of charge transfer. For clarity, hydrogen atoms were omitted from the analysis due to their negligible contribution to the electronic transitions of interest. The TDM plots are presented in Fig. 6. In these two-dimensional maps, the atomic indices are plotted on the *x*- and *y*-axes, while the color scale (right *y*-axis) represents the magnitude of the transition density between atom pairs. The color intensity directly correlates with the degree of electron–hole correlation: redregions indicate a strong, localized electron–hole pair (high co-localization of the excited electron and the resulting hole). Blue regions indicate weak or negligible correlation, and intermediate colors represent regions of moderate or delocalized transition activity. Bright spots on the diagonal of the plot correspond to local excitations within a specific fragment (donor or acceptor), while off-diagonal features signify charge-transfer (CT) character between different molecular regions. The TDM plot for R shows a predominantly faded diagonal, which may indicate weak local excitation and minimal charge transfer from the donor to the acceptor region. In all designed molecules, the molecular framework was segmented into donor, π-linker, and acceptor fragments to explicitly track the charge transfer pathway. The analysis predicts that both the donor core and the terminal acceptor groups are critically involved in the exciton dynamics. The TDM plot for BD4 is distinctive, displaying a bright, continuous diagonal line extending from the bottom-left (donor) to the top-right (acceptor). This result suggests strong electron–hole coherence and efficient intramolecular charge transfer across the entire π-system. This may be the direct consequence of the strong electron-withdrawing sulfonyl chloride (–SO_2_Cl) end group. The TDM plots for BD1, BD2, BD3, and BD5 predict less intense diagonal features compared to BD4, correlating with reduced electron–hole localization and weaker charge transfer character. BD1 exhibits a moderately faded diagonal, while BD2, BD3, and BD5 show even less pronounced localization. The TDM analysis supports our molecular design strategy. The incorporation of a methyl thieno[3,4-*b*]thiophene-2-carboxylate π-linker, coupled with strong terminal acceptors, successfully enhances intramolecular charge transfer to the acceptor region. The computed TDM profile of BD4, characterized by strong diagonal coherence and effective charge separation, predicts its excellent potential for device performance, positioning it as a superior candidate for the development of next-generation, high-efficiency organic solar cells.

**Fig. 6:**
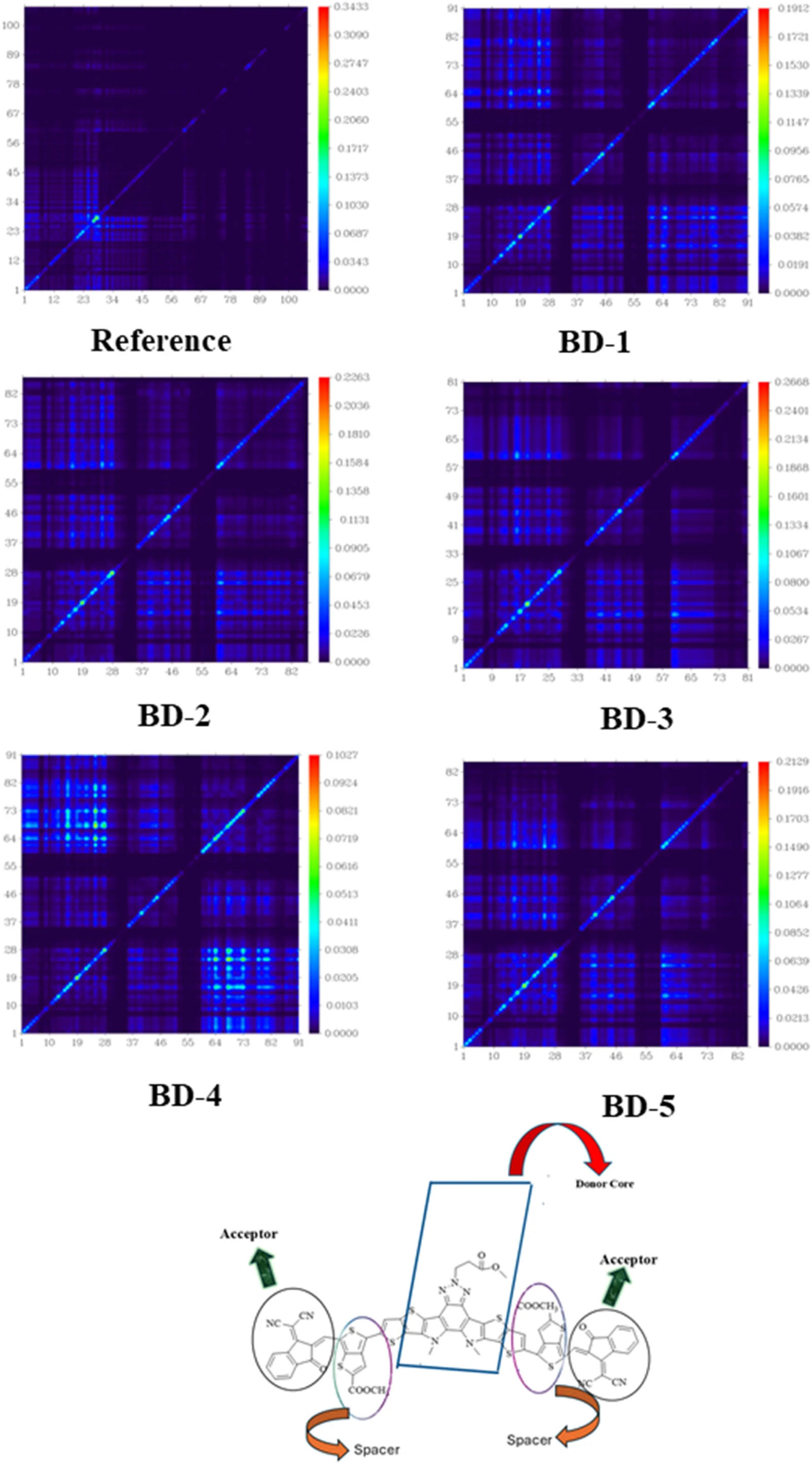
TDM plots for R and BD1–BD5.

### 3.8 Reduced density gradient analysis

Non-covalent interaction (NCI) analysis is a crucial tool for elucidating the structural coherence and stability of molecular materials, which may directly impact their performance in optoelectronic devices such as LEDs and solar cells.^34^ NCI plots visualize intermolecular forces including hydrogen bonding, van der Waals interactions, and steric repulsion, based on the reduced density gradient (RDG) and the sign of the second eigenvalue of the electron density Hessian (sign(*λ*_2_)*ρ*). The NCI isosurface plots for the designed molecules are presented in Fig. 7. In these visualizations, different interaction types are distinguished by color: blue isosurfaces represent strong, attractive non-covalent interactions, such as hydrogen bonds. Green isosurfaces indicate weaker, dispersion-dominated interactions, primarily van der Waals forces, and red isosurfaces signify strong, repulsive steric clashes. All designed molecules exhibit more pronounced blue regions (indicative of hydrogen bonding) compared to the reference molecule (R). This suggests enhanced intramolecular charge transfer (ICT) character, which is favorable for charge separation. Molecules BD5, BD2, and BD3 display more extensive and scattered green isosurfaces, which is conducive to favorable π–π stacking. In contrast, BD1 and BD4 show a relatively more confined distribution of green regions. BD2 and BD3 exhibit increased red isosurfaces, which may indicate greater steric hindrance. This structural congestion can disrupt optimal π-conjugation and can adversely affect the short-circuit current density (*J*_SC_). A comparative analysis of the NCI plots reveals that BD5 and BD1 present the most favorable balance of interactions. They feature strong hydrogen bonding (intense blue), well-localized van der Waals forces (focused green), and minimal steric repulsion (negligible red). This optimized interaction profile supports both efficient intramolecular charge transfer and favorable solid-state packing. The NCI analysis may help in the prediction of structural rationale for the observed electronic trends. The introduction of strong electron-withdrawing groups (EWGs) at the terminal positions shifts the sign(*λ*_2_)*ρ* peaks to more negative values, correlating with the modulation of interaction strengths. The dense blue regions associated with these EWGs can directly correlate with the enhanced ICT and reduced band gaps discussed in previous sections.

**Fig. 7:**
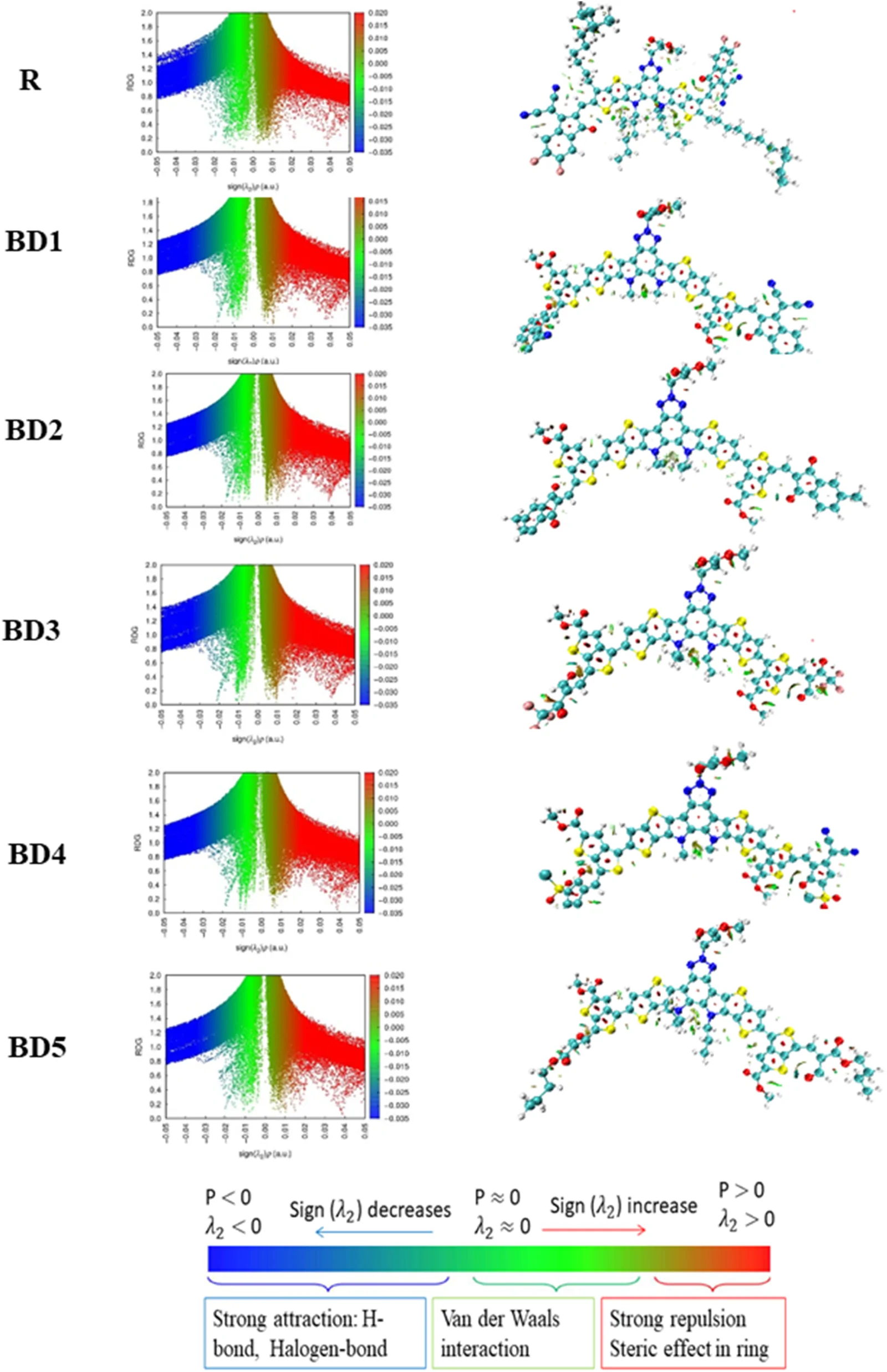
Graphical illustration of NCI for R and designed molecules.

### 3.9 Dipole moment

The dipole moment (*µ*) is a key descriptor of molecular polarity, stability, and charge transport behavior, reflecting the degree of spatial charge separation within a molecule. In organic solar cells, a well-defined dipole facilitates effective charge segregation and transport, which are critical for high device performance. A higher dipole moment is generally associated with beneficial properties such as increased solubility, improved thin-film morphology, and enhanced photovoltaic efficiency.^35^ The dipole moments for all molecules were calculated at the DFT/MPW1PW91/6-311G(d,p) level of theory, and the complete dataset is provided in SI Table S2 and visualized in SI Fig. S7. Among the designed derivatives, BD4 exhibits the highest dipole moment in both the ground (S_0_) and excited (S_1_) states. The calculated values are as follows: ground state (S_0_): BD4 (13.11 D) > BD3 (7.81 D) > BD5 (4.62 D) > BD1 (3.61 D) > BD2 (2.44 D). In excited state, these values are as: (S_1_): BD4 (14.77 D) > BD3 (9.36 D) > BD5 (6.13 D) > BD1 (4.02 D) > BD2 (5.51 D). For comparison, the reference molecule (R) has dipole moments of 10.62 D (S_0_) and 13.2 D (S_1_), confirming that BD4 surpasses even the reference in polarity. The significant increase in dipole moment from the ground to the excited state in BD4 is particularly notable, indicating a pronounced charge redistribution upon photoexcitation.

This elevated polarity is advantageous for several reasons: it promotes molecular crystallinity, optimizes solid-state packing, and creates a strong internal electric field that drives the separation and transport of photogenerated charge carriers (holes and electrons). Consequently, these properties contribute to a higher fill factor (FF) and overall device performance. The outstanding dipole moment of BD4 underscores its excellent potential as a photoactive material for next-generation organic solar cells.

### 3.10 Light harvesting efficiency

The light-harvesting efficiency (LHE) is a critical parameter that measures a solar cell's effectiveness in absorbing incident light and generating electrical energy. It directly influences the photocurrent, and therefore the short-circuit current density (*J*_SC_) and power conversion efficiency (PCE). The LHE of a molecular absorber is determined by its oscillator strength (*f*), which quantifies the probability of the electronic transition involved in photon absorption. Consequently, a higher oscillator strength leads to a higher LHE. The LHE is computed from the oscillator strength using eqn (3)^21^ are presented in Table 1 and S1 SI for both solvent and gas phases, respectively.

3 LHE = 1 − 10^−*f*^

As indicated by eqn (3), the light-harvesting efficiency (LHE) is directly related to the oscillator strength (*f*). In agreement with the high oscillator strengths, all designed molecules exhibit significantly enhanced LHE compared to the reference (R). The calculated LHE values follow the order in the solvent phase as: BD2 (0.998) = BD1 (0.998) > BD3 (0.996) = BD5 (0.996) > BD4 (0.942) > R (0.31). In the gas phase, the following order is observed: BD2 (0.997) > BD1 (0.996) > BD3 (0.994) = BD5 (0.994) > BD4 (0.939) > R (0.706). BD2 shows the highest LHE in both phases, closely followed by BD1. The elevated LHE directly results from the high oscillator strength, which maximizes the absorption profile of the molecules. The near-unity LHE values (>0.99) for BD1, BD2, BD3, and BD5 indicate exceptional theoretical light-harvesting capability. The LHE is a critical parameter as it is directly linked to the short-circuit current density (*J*_SC_), a primary determinant of power conversion efficiency. This relationship is expressed in eqn (4). The higher LHE values of the designed molecules, therefore, predict a substantially higher *J*_SC_ and, consequently, improved device performance compared to the reference.4
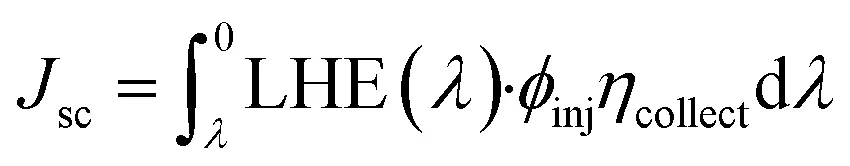
Here, *φ*_inj_ and *η*_collect_ signify charge injection and collection efficiency. Consequently, the enhanced LHE of the designed molecules directly contributes to a higher theoretical short-circuit current density (*J*_SC_), as *J*_SC_ fundamentally depends on the efficiency of photon absorption across the solar spectrum. While other factors (such as charge carrier mobility and recombination) also influence the practical *J*_SC_, the significantly elevated LHE values, particularly for BD1 and BD2, demonstrate a superior intrinsic capacity for light harvesting. This result underscores the strong potential of these materials to improve incident photon absorption and, by extension, the overall performance of organic solar cells (OSCs).

### 3.11 Chemical reactivity

The global reactivity descriptors for the tailored molecules were calculated at the DFT/MPW1PW91/6-311G(d,p) level. These included chemical hardness (*η*), softness (*S*), electronegativity (*χ*), chemical potential (*µ*), and electrophilicity (*ω*). Analysis of these parameters gives essential information about kinetic stability, chemical reactivity, donor–acceptor interaction strength, polarizability, and structural stability.^36,37^ All these parameters are calculated by using eqn (5)–(9), and the computed values for all parameters are compiled in Table 4.

5 *µ* = *E*_HOMO_ + *E*_LUMO_/2

6 *η* = (*E*_HOMO_ − *E*_LUMO_)/2

7 *S* = 1/2*η*

8 *ω* = *µ*^2^/2*η*

9 *χ* = −*µ*

**Table 4:** Chemical softness (*S*), chemical hardness (*η*), chemical potential (*µ*), electronegativity (*χ*), and electrophilicity (*ω*) of the modified molecule were determined through DFT/MPW1PW91/6-311G (d,p) level of theory

Molecules	HOMO (eV)	LUMO (eV)	*E*_g_ (eV)	*µ* (eV)	*η* (eV)	*ω* (eV)	*S* (eV)^−1^	*χ* (eV)
R	−5.60	−3.49	2.11	−4.55	1.01	10.29	0.49	4.55
BD1	−5.49	−3.53	1.96	−4.51	0.98	10.38	0.51	4.51
BD2	−5.57	−3.17	2.19	−4.37	1.09	8.76	0.46	4.37
BD3	−5.58	−3.49	2.40	−4.54	1.20	8.59	0.21	4.54
BD4	−5.64	−3.98	1.66	−4.81	0.83	13.94	0.60	4.81
BD5	−5.42	−3.18	2.24	−4.30	1.12	8.84	0.45	4.30

Chemical potential (*µ*) is a key global reactivity descriptor. In the context of photovoltaics, it relates to the change in system energy upon the addition of an electron, which influences the open-circuit voltage (*V*_oc_). Among the designed molecules, BD4 exhibits the highest chemical potential (*µ* = −4.81 eV), surpassing both the reference (R) and the other derivatives (BD1: −4.51 eV, BD2: −4.31 eV, BD3: −4.54 eV, BD5: −4.30 eV). This elevated *µ* is a direct consequence of the strong electron-withdrawing sulfonyl chloride (–SO_2_Cl) terminal group, which modifies the frontier molecular orbital (FMO) energies and the distribution of electron–hole pairs. The higher chemical potential of BD4 is favorable for enhanced charge extraction, improved charge transfer, and a higher theoretical *V*_oc_.

Chemical softness (*S*) and hardness (*η*) are inversely related descriptors of molecular reactivity and stability, as confirmed by the data in Table 5. A high *S* indicates a lower resistance to electron flow, facilitating charge transfer. BD4 shows the highest chemical softness and, correspondingly, the lowest hardness among the series. This combination suggests that BD4 is both highly chemically reactive (facile electron transfer) and structurally stable, validating the success of the π-spacer insertion strategy in promoting electron mobility.

**Table 5:** Estimated open-circuit voltage (*V*_oc_) and normalized *V*_oc_

Molecules	*V*_oc_	Normalized (*V*_oc_)
R	1.68 V	65.00
BD1	1.64 V	63.46
BD2	2.00 V	77.39
BD3	1.68 V	65.00
BD4	1.19 V	46.04
BD5	1.99 V	77.00

Electrophilicity (*ω*) and electronegativity (*χ*) quantify the tendency of a molecule to accept electrons and attract electron density, respectively. BD4 also possesses the highest values for both *ω* (13.94 eV) and *χ* (4.81 eV) compared to the other molecules. These elevated values indicate a strong driving force for intramolecular charge transfer (ICT) from the donor to the acceptor moiety. Collectively, the global reactivity descriptors for BD4, such as high chemical potential, softness, electrophilicity, and electronegativity, confirm that the strategic introduction of a strong electron-acceptor group effectively reduces the HOMO–LUMO gap, increases chemical reactivity, and enhances ICT. These properties are crucial for the formation of an efficient active layer in organic solar cells, as they promote efficient light absorption, charge generation, and transport.

### 3.12 Hole–electron transport

Analyzing molecular excitation dynamics is essential for understanding charge generation and transport.^38^ Multiwfn software was employed to visualize the spatial distribution of electron density changes upon excitation, providing a direct map of charge transfer pathways,^39^ which is depicted in Fig. 8. These maps illustrate a consistent mechanism across all designed molecules (BD1–BD5). Upon photoexcitation, holes (depletion of electron density) are generated primarily within the donor region. Concurrently, a corresponding increase in electron density is observed across the π-conjugated linker and localizes on the terminal electron-withdrawing acceptor groups (*e.g.*, –C(CN)_2_ in BD1, –SO_2_Cl in BD4). This visualization confirms that the π-spacer acts as an effective bridge, facilitating the transfer of electron density from the donor core to the acceptor terminals. This clear spatial separation of holes (donor) and electrons (acceptor) is indicative of efficient intramolecular charge transfer (ICT). The balanced overlap of the hole and electron distributions along the molecular axis is vital for minimizing charge recombination. The localization of increased electron density on strong acceptor groups like –CN and –SO_2_Cl underscores their role in stabilizing the separated charge. Collectively, these observations confirm that all designed molecules exhibit effective, spacer-mediated charge transfer from donor to acceptor, a fundamental requirement for high-performance organic photovoltaic materials.

**Fig. 8:**
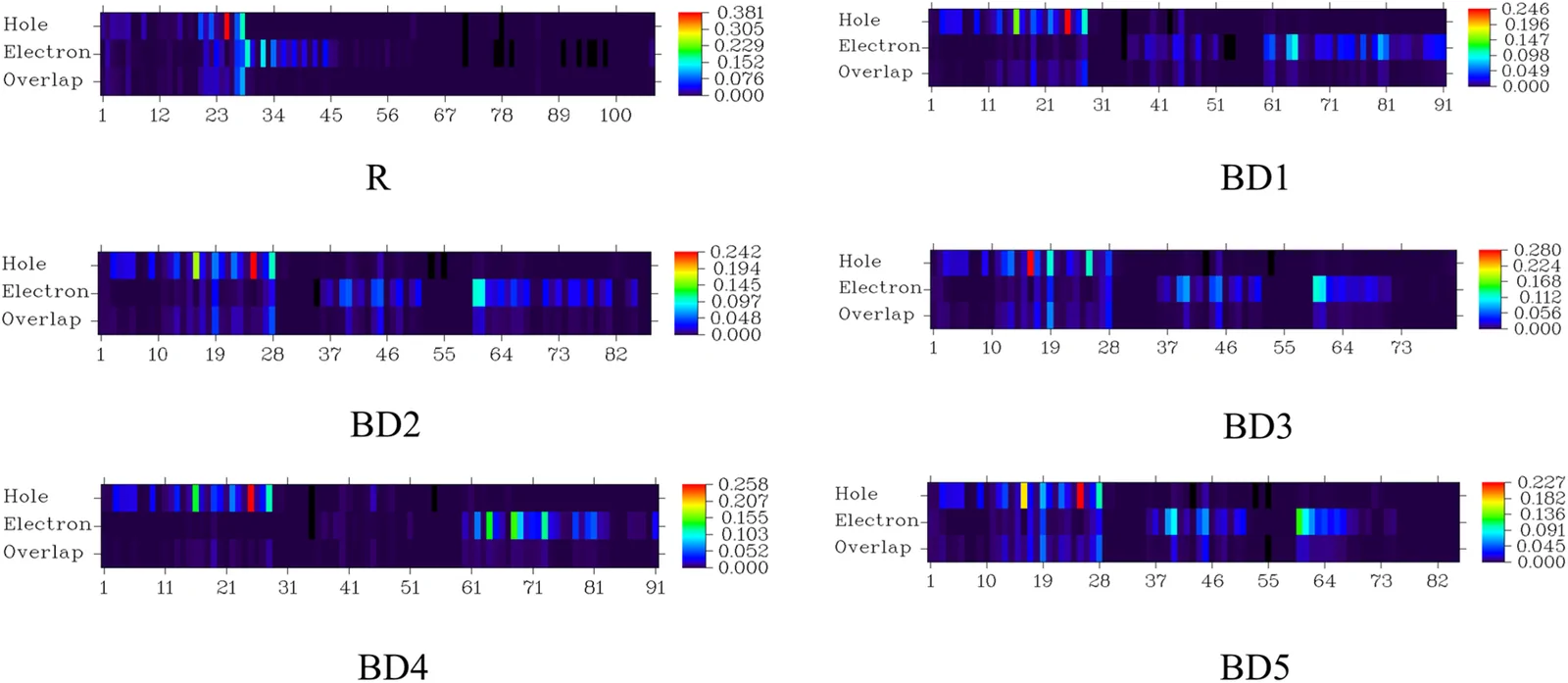
Pictorial representation of hole–electron transport in reference and modified molecules (BD1–BD5).

### 3.13 Open-circuit voltage

The open-circuit voltage (*V*_oc_) is a critical parameter for determining the power conversion efficiency (PCE) of non-fullerene acceptor-based solar cells. *V*_oc_ is defined as the maximum voltage available from the device when the net current flow is zero. Its value is influenced by several factors, including the exciton binding energy, the alignment of molecular energy levels, and the incident light intensity.^40^ Theoretically, *V*_oc_ can be estimated using the Scharber model,^41^ and a higher *V*_oc_ directly contributes to a higher overall PCE. Scharber model provides a meaningful screening-level approximation for our designed NFAs. Eqn (10) is used to calculate *V*_oc_ as:10
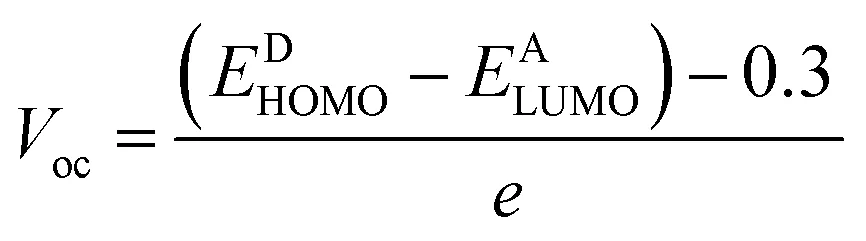
where *E*^D^_HOMO_ indicates the HOMO level of the donor, *E*^A^_LUMO_ refers to the LUMO energy level of the acceptor molecule, and *e* is the elementary charge on a molecule, which is taken as 1. Meanwhile, 0.3 is an empirical factor account for energy losses because of charge recombination. These energy losses vary depending on the donor–acceptor combination, device architecture, and film morphology. Therefore, estimated *V*_oc_ and PCE are considered idealized theoretical estimates. The theoretical open-circuit voltage (*V*_oc_) for the reference and designed acceptor molecules (BD1–BD5) was calculated based on their pairing with the PBFTT polymer donor visualized in Fig. 9 and tabulated in Table 5. This pairing is considered due to the favorable alignment of their energy levels. The HOMO and LUMO energies of PBFTT are −5.47 eV and −3.56 eV, respectively. The *V*_oc_ for a donor–acceptor blend is primarily determined by the energy difference between the HOMO of the donor (PBFTT) and the LUMO of the acceptor. The computed *V*_oc_ values for the BD1–BD5 series paired with PBFTT are as follows: BD1 (1.64 V), BD2 (2.00 V), BD3 (1.88 V), BD4 (1.19 V), and BD5 (1.00 V). The data indicate that BD2 and BD5 yield the highest *V*_oc_, while BD4 gives the lowest. The high *V*_oc_ of BD2 and BD5 is attributed due to their higher LUMO level of the acceptor. According to the Scharber model, the energy difference between the HOMO of the donor and the LUMO of the acceptor increases because of the higher-lying acceptor, which ultimately enhances the *V*_oc_. In contrast, BD4 exhibits the lowest LUMO energy level, which contributes to lower *V*_oc._

**Fig. 9:**
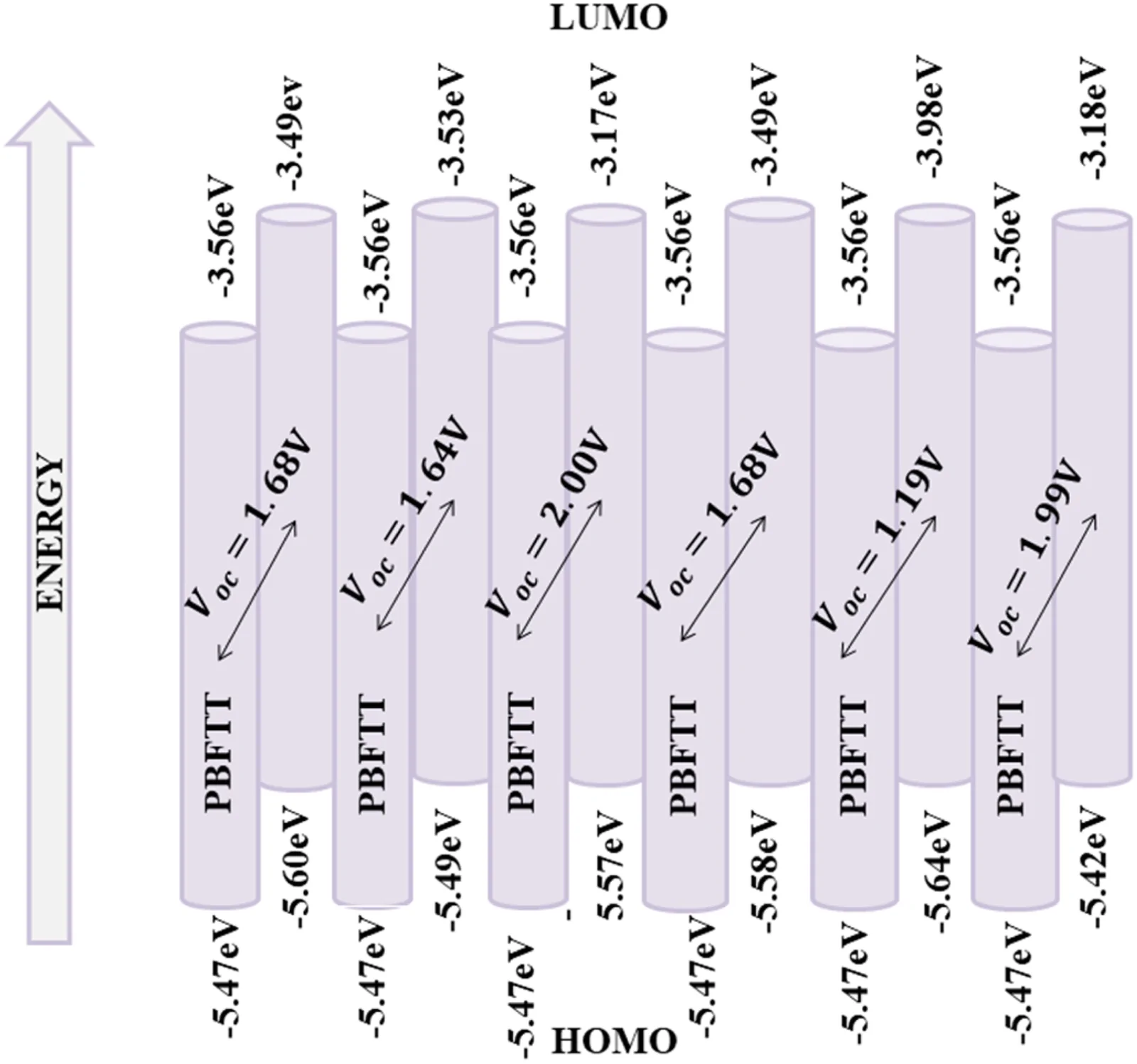
Elucidation of *V*_oc_ values for R and all modified molecules (BD1–BD5).

BD4 exhibits the lowest *V*_oc_ and, as noted in other sections, a lower predicted short-circuit current density (*J*_SC_). This highlights the complex interplay of factors governing device performance. While BD4 has the narrowest band gap (favorable for absorption and *J*_SC_), its *V*_oc_ is limited by the specific energy offset with PBFTT. The polymer donor PBFTT was chosen because of its appropriate energy level alignment with the acceptor molecule and used as a benchmarked donor in computational studies. The use of PBFTT also allows meaningful comparison of relative photovoltaic potential in modified acceptors. Su *et al.*^42^ fabricated an ST-OSC based on a PBFTT donor and IT-4Cl acceptor, which exhibit well-aligned energy levels and absorption spectra in the near-IR region. Furthermore, parameters such as exciton binding energy and the driving force for charge separation, which are influenced by the donor–acceptor energy offset, also significantly impact *J*_SC_. This demonstrates that optimizing overall power conversion efficiency requires balancing a high *V*_oc_ (large energy offset) with a high *J*_SC_ (low band gap and efficient charge generation).

### 3.14 Fill factor and short-circuit current

The fill factor (FF) is a key dimensionless parameter for evaluating solar cell efficiency. It describes the “squareness” of the current–voltage (*J*–*V*) curve, quantifying how effectively the cell converts incident light into electrical power. A high FF, often correlated with a high open-circuit voltage (*V*_oc_), directly contributes to a higher PCE. The FF for the reference and designed molecules was calculated using the ideal Shockley–Queisser detailed-balance limit equation given in eqn (11).^35^11
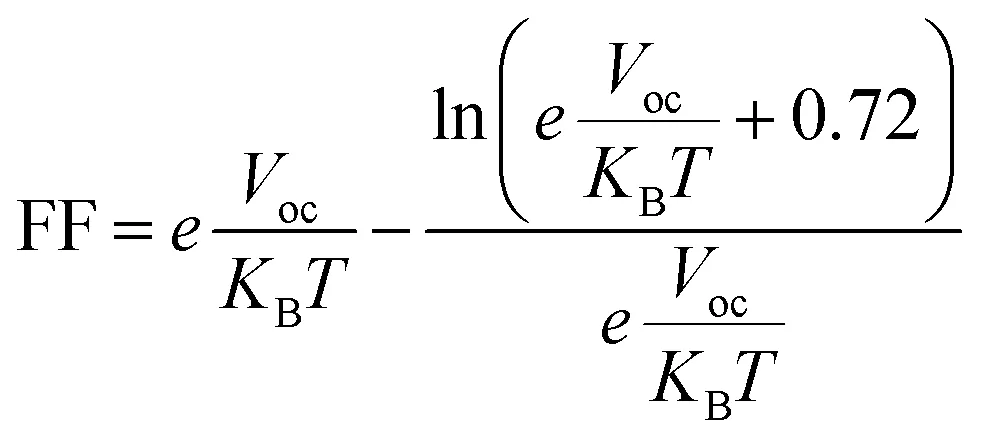
In this equation, normalize *V*_oc_ is calculated by 
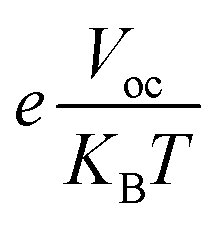
 and then FF is computed by using normalized 
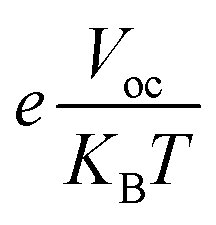
 where *K*_B_ is Boltzmann's constant (8.61733 × 10^−5^ eV K^−1^). This approach yields the FF, which is close to unity and overestimates the achievable experimental FF. This equation assumes ideal device behaviour by neglecting charge recombination losses and series resistance, which results in overestimation of FF. The calculated fill factor (FF) values for the designed molecules are shown in Table 6, as follows: BD1 (0.919), BD2 (0.932), BD3 (0.921), BD4 (0.897), and BD5 (0.931). All modified molecules exhibit enhanced FF values compared to the reference structure. The FF trend increases in the order: BD4 < BD1 < BD3 < BD5 < BD2. This indicates that BD2 and BD5 possess the highest fill factors within the series. The improved FF in the designed molecules can be attributed to two key factors linked to their electronic structure: reduced reorganization energy, as lower reorganization energy facilitates more efficient charge transport, minimizing energy losses and contributing to a higher FF. A high fill factor is a direct indicator of efficient charge extraction and minimal electrical losses in a solar cell. Therefore, the higher FF values of BD2 and BD5 suggest they are particularly promising candidates for the development of next-generation high-performance organic solar cells. The power conversion efficiency of a solar cell is directly dependent on the short-circuit current density (*J*_SC_), which quantifies the maximum photocurrent the device can generate under illumination. The *J*_sc_ of all molecules is calculated by using eqn (12):^43^12
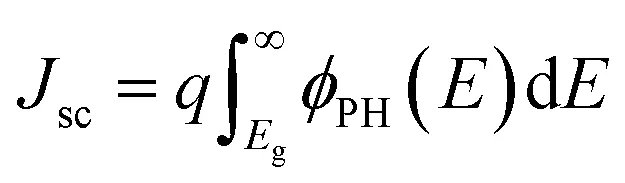
Here, *q* and *ϕ*_PH_ represent the elementary charge and photon flux density, respectively. This approximation provides an idealized upper-limit estimate of photocurrent generation by assuming complete absorption of incident photons. Therefore, estimated *J*_SC_ does not deal with device-level effects such as film morphology, charge recombination, and optical losses. As indicated by eqn (12), the short-circuit current density (*J*_SC_) exhibits an inverse relationship with the optical band gap. This relationship is clearly demonstrated by the results for the designed molecules. The molecule with the narrowest band gap, BD4, achieves the highest calculated theoretical *J*_SC_ of 22.0 mA cm ^2^ as given in Table 6. The *J*_SC_ values for the series follow the decreasing order: BD4 (22.0 mA cm^−2^) > BD1 (15.0 mA cm^−2^) > BD2 (9.0 mA cm^−2^) > BD5 (8.0 mA cm^−2^) > BD3 (7.0 mA cm^−2^). The complete set of *J*_SC_ values, including that of the reference molecule (R), is provided in Table 6. The higher suggested *J*_SC_ value of BD4 is a direct consequence of its reduced band gap, which extends its absorption profile further into the red region of the solar spectrum. This allows BD4 to harvest a greater number of photons, thereby generating a higher photocurrent. The observed trend supports the critical design principle that reducing the HOMO–LUMO gap is an effective strategy for boosting the current-generating capability of non-fullerene acceptor materials in organic solar cells.

**Table 6:** Estimated value of Fill Factor (FF), short-circuit current (*J*_sc_), and power conversion efficiency (PCE)

Molecules	FF	*J*_sc_ (mA cm^−2^)	PCE	Experimental PCE
R	0.921	11	17.02%	18.33%
BD1	0.919	15	22.61%	
BD2	0.932	9	16.78%	
BD3	0.921	7	10.83%	
BD4	0.897	22	23.48%	
BD5	0.931	8	14.82%	

### 3.15 Power conversion efficiency

Power conversion efficiency (PCE) is a critical performance parameter, defined as the ratio of electrical power output to the incident solar power input. The theoretical PCE for the designed molecules is evaluated using eqn (13).^44^ Here, *J*_SC_, *V*_oc_, and FF were estimated by established empirical models. This expression is widely used in computational screening of photovoltaic materials because it allows the evaluation of the photovoltaic potential of a molecule.13
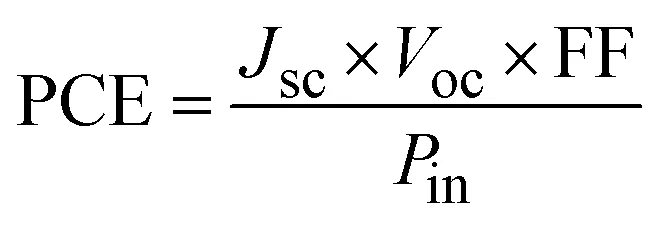


The calculated idealized theoretical estimates of power conversion efficiencies (PCEs) for the designed molecular acceptors are presented in Fig. 10 and detailed in Table 6. Among all designed molecules, BD4 achieves the highest predicted theoretical PCE of 23.48%, indicating superior potential for converting sunlight into electricity. It is followed by BD1 with a PCE of 22.61%. The complete theoretical PCE ranking is as follows: BD4 (23.48%) > BD1 (22.61%) > BD2 (16.78%) > BD5 (14.82%) > BD3 (10.83%). All designed molecules show enhanced photovoltaic characteristics compared to the reference molecule (R). The superior performance of BD4 and BD1 is particularly noteworthy. Their high PCE values are primarily driven by their combination of high theoretical *J*_SC_ values, resulting from their low band gaps and strong absorption, along with favorable open-circuit voltage (*V*_oc_) and fill factor (FF) values achieved through optimal energy level alignment and efficient charge transport. Therefore, this computational study identifies BD4 and BD1 as the most promising candidates for the experimental fabrication of high-efficiency organic solar cells (OSCs). Their outstanding predicted performance stems from a well-balanced optimization of key photovoltaic parameters, validating the success of the applied molecular design strategy.

**Fig. 10:**
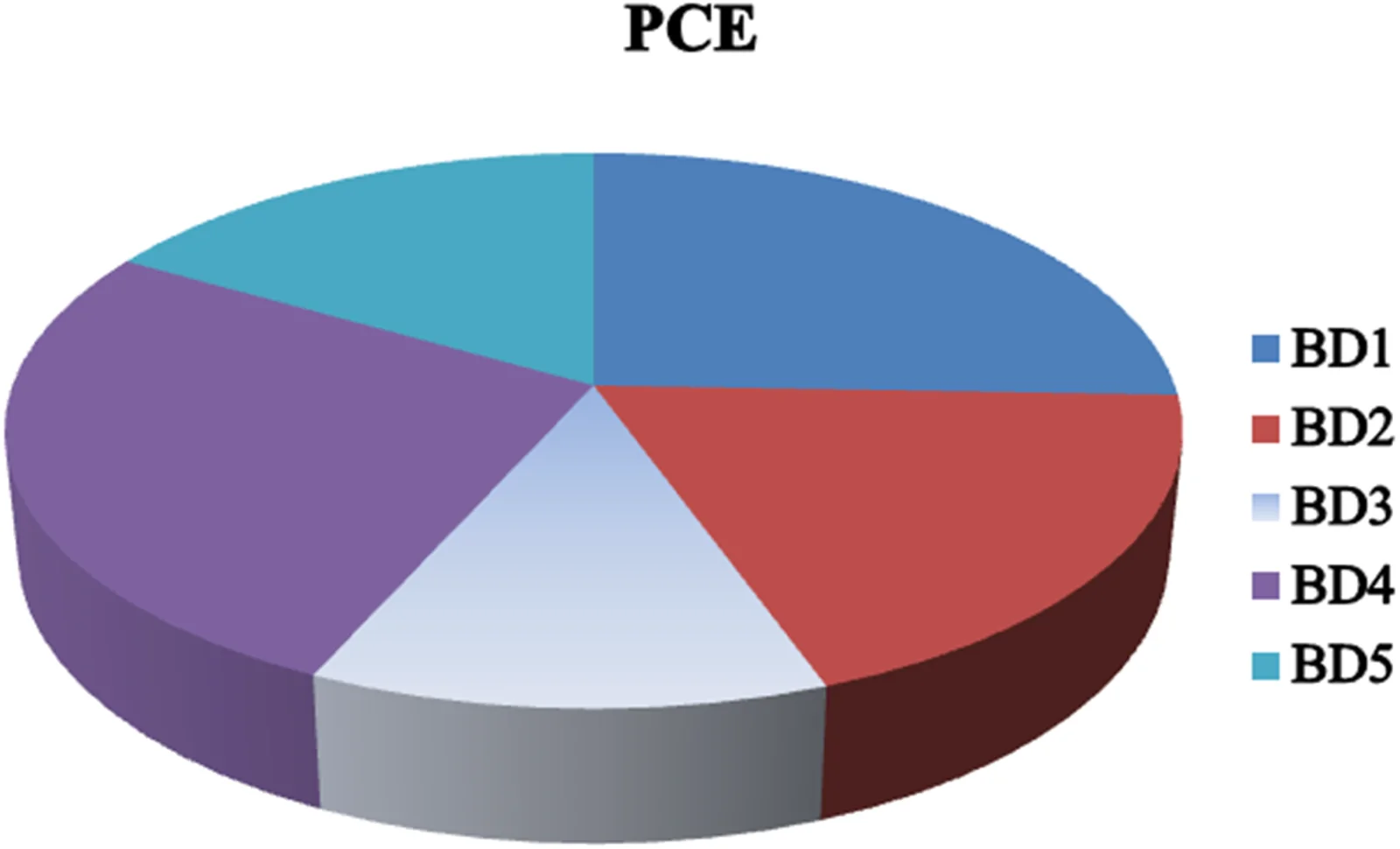
Illustration of PCE for BD1–BD5 molecules.

It is noted that PCE is calculated by using an empirical Scharber-type relationship based on calculated *V*_oc_, *J*_SC_, and assumed fill factor. This is a preliminary computational approach for screening of organic photovoltaic devices. However, it fails to capture real-world device performance, including film morphology, charge recombination, and fabrication method. Consequently, estimated PCE should be interpreted as idealized benchmarks for molecular comparison rather than predicting physical device efficiency. Fig. 10 shows the calculated PCE values of our designed molecules in pie chart. The estimated theoretical PCE of 29.97% was reported by Hussain *et al.*^45^ in 2024 for NFA *via* computational study of designed molecules (AY1–AY8).

## 4 Conclusion

This computational study demonstrates a combined molecular engineering approach *via* incorporation of a π-linker with terminal acceptor group modification for advancing benzotriazole (BZ)-based non-fullerene small-molecule acceptors (NF-SMAs). Through systematic density functional theory (DFT) and time-dependent DFT (TD-DFT) investigations, we designed and evaluated five novel derivatives (BD1–BD5) from a high-performance reference (BZE-31).

The engineered methyl thieno[3,4-*b*]thiophene-2-carboxylate π-linker, coupled with strong electron-withdrawing terminal groups, fundamentally enhanced the optoelectronic attributes. All designed molecules exhibited a pronounced bathochromic shift (*λ*_max_ = 690–939 nm in chloroform) and significantly reduced optical band gaps (1.66–2.24 eV) compared to the reference. Comprehensive analyses including frontier molecular orbitals, density of states, transition density matrices, and global reactivity descriptors consistently validated enhanced intramolecular charge transfer, favorable spatial charge separation, and optimized energy level alignment.

Among the series, BD4 (functionalized with a 4-methylene-5-oxocyclopent-1-ene-1-sulfonyl chloride end group) emerged as the most promising candidate. It achieved the narrowest band gap (1.66 eV), the most red-shifted absorption (939 nm), the lowest exciton binding energy (0.31 eV), and the highest calculated short-circuit current density (*J*_sc_ ≈ 22 mA cm^−2^). BD1 (with a malononitrile end group) also demonstrated superior performance. When theoretically paired with the PBFTT donor, these molecules yielded outstanding theoretical power conversion efficiencies (PCEs) of 23.48% for BD4 and 22.61% for BD1 with the adopted theoretical screening model. This exceptional performance originates from an optimal balance of a high *J*_sc_ (enabled by enhanced light-harvesting), a favorable open-circuit voltage, and an excellent fill factor facilitated by efficient charge transport. This work provides a validated computational blueprint that deciphers the structure–property relationships in BZ-based NF-SMAs. It conclusively identifies BD4 and BD1 as prime synthetic targets, offering a clear and actionable pathway for the experimental realization of next-generation, high-efficiency organic solar cells.

## Conflicts of interest

None of the authors have any conflicts of interest to declare.

## Supplementary Material

RA-016-D6RA05880A-s001

## Data Availability

Data will be provided upon reasonable request from the corresponding author. Supplementary information (SI) is available. See DOI: https://doi.org/10.1039/d6ra05880a.
