# Supplementary material for: From molecular design to high performance: interpreting the combined effect of π-linkers and acceptor groups in benzotriazole-based non-fullerene acceptors
Source: RSC Adv. 2026 Oct 19;16(48):64141–57. doi: 10.1039/d6ra05880a (PMC13678501; doi:10.1039/d6ra05880a)
Supplement: Supplementary file 1 [file RA-016-D6RA05880A-s001.pdf]

Supplementary Information (SI) for RSC Advances.
This journal is © The Royal Society of Chemistry

From Molecular Design to High Performance: Interpreting the Combined Effect of π-Linkers and Acceptor Groups in Benzotriazole-Based Non-Fullerene Acceptors

Komal Ateeq[1], Muhammad Kamran Shehzad[2], Yasir Altaf*[1], Maaz Ur Rehman[1], Rabia Bassran[1], Muhammad Farhan Waris[1], Fahim Ahmed[3], Farasat Haider[4], Basit Ali[5]*

[1] *Department of Chemistry, Division of Science and Technology, University of Education, Lahore 54770, Pakistan*

[2] *School of Materials Science and Engineering, Hefei University of Technology, Hefei 230009, China*

[3] *Department of Physics, University of Sargodha, 40100 Sargodha, Pakistan*

[4] *International Graduate School of Nanoscience and Technology, College of Interdisciplinary and Integrative Studies, Chulalongkorn University, Bangkok 10330, Thailand*

[5] *Department of Chemistry and Materials Science, School of Chemical Engineering, Aalto University, P.O. Box 16100, FI-00076 Aalto, Finland*

Corresponding authors:

Yasir Altaf (yasir.altaf@ue.edu.pk)

Basit Ali (basit.ali@aalto.fi)

Supplementary Information

Table S1: Calculated values of absorption profile (λ_{max}), Oscillator strength (*f*), Light Harvesting Efficiency (LHE), Excitation energy (E_x), molecular orbital (MO) assignment, exciton binding energy (E_b) from HOMO-LUMO (H→L), and band gap (E_g) in the gas phase.

Molecules	λ_{max} (nm)	*f*	LHE	E_x (eV)	Major MO assignment	E_b (eV)	E_g (eV)
R	770	0.05	0.706	1.61	H→L 98%	0.40	2.01
BD1	738	2.49	0.996	1.68	H→L 94.3%	0.34	2.02
BD2	668	2.53	0.997	1.85	H→L 94.4%	0.37	2.22
BD3	685	2.24	0.994	1.81	H→L 96.8%	0.32	2.13

BD4	826	1.22	0.939	1.49	H→L 92.7%	0.31	1.81
BD5	653	2.22	0.994	1.89	H→L 95.6%	0.35	2.25

Table S2: Dipole moment of R and BD1-BD5 at ground state (μ_g) and excited state (μ_e) computed by MPW1PW91/6-311G (d, p) level of theory

Molecules	μ_e (Debye)	μ_g (Debye)	μ_e - μ_g (Debye)
R	13.20	10.63	2.57
BD1	4.01	3.61	0.4
BD2	3.51	2.44	1.07
BD3	9.36	7.81	1.55
BD4	14.78	13.11	1.67
BD5	6.13	4.62	1.51

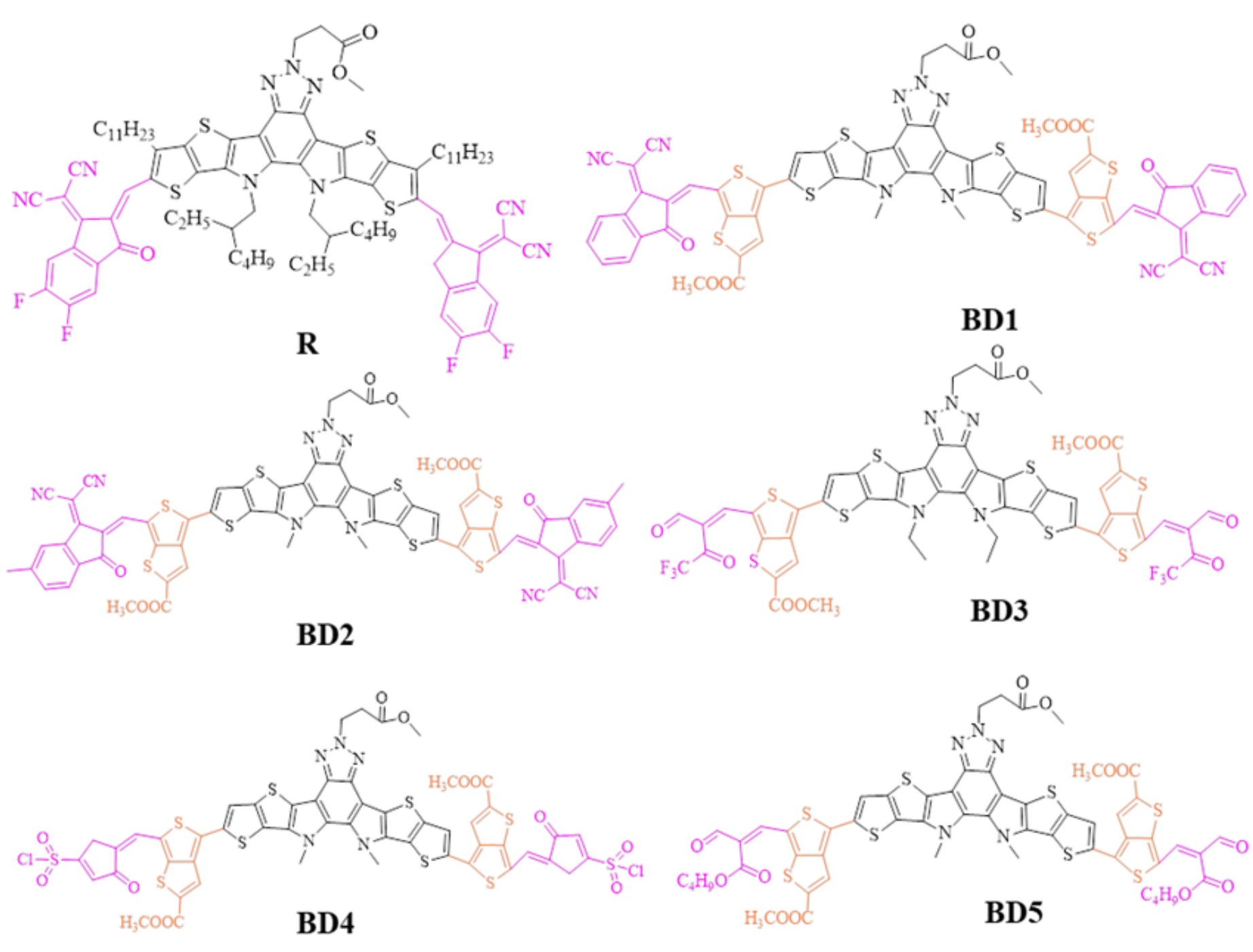

Supplementary figure S1: 2D structure of designed molecules

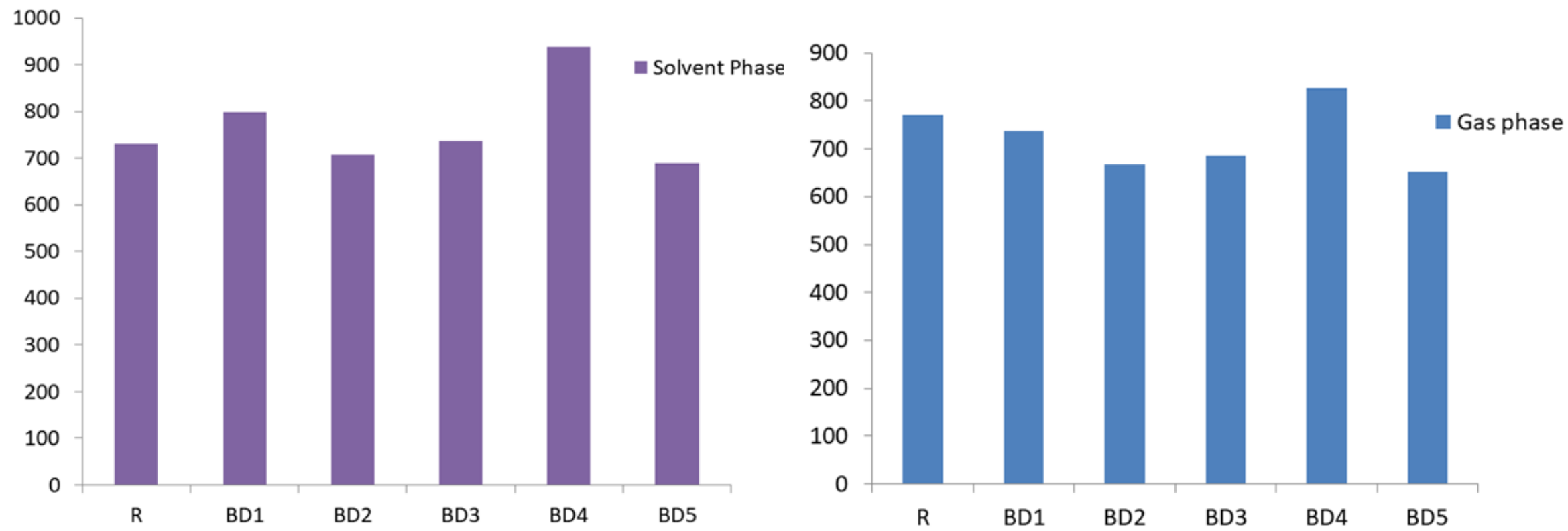

S2: Absorption wavelength of all modified molecules in both the solvent (chloroform) and the gas phases

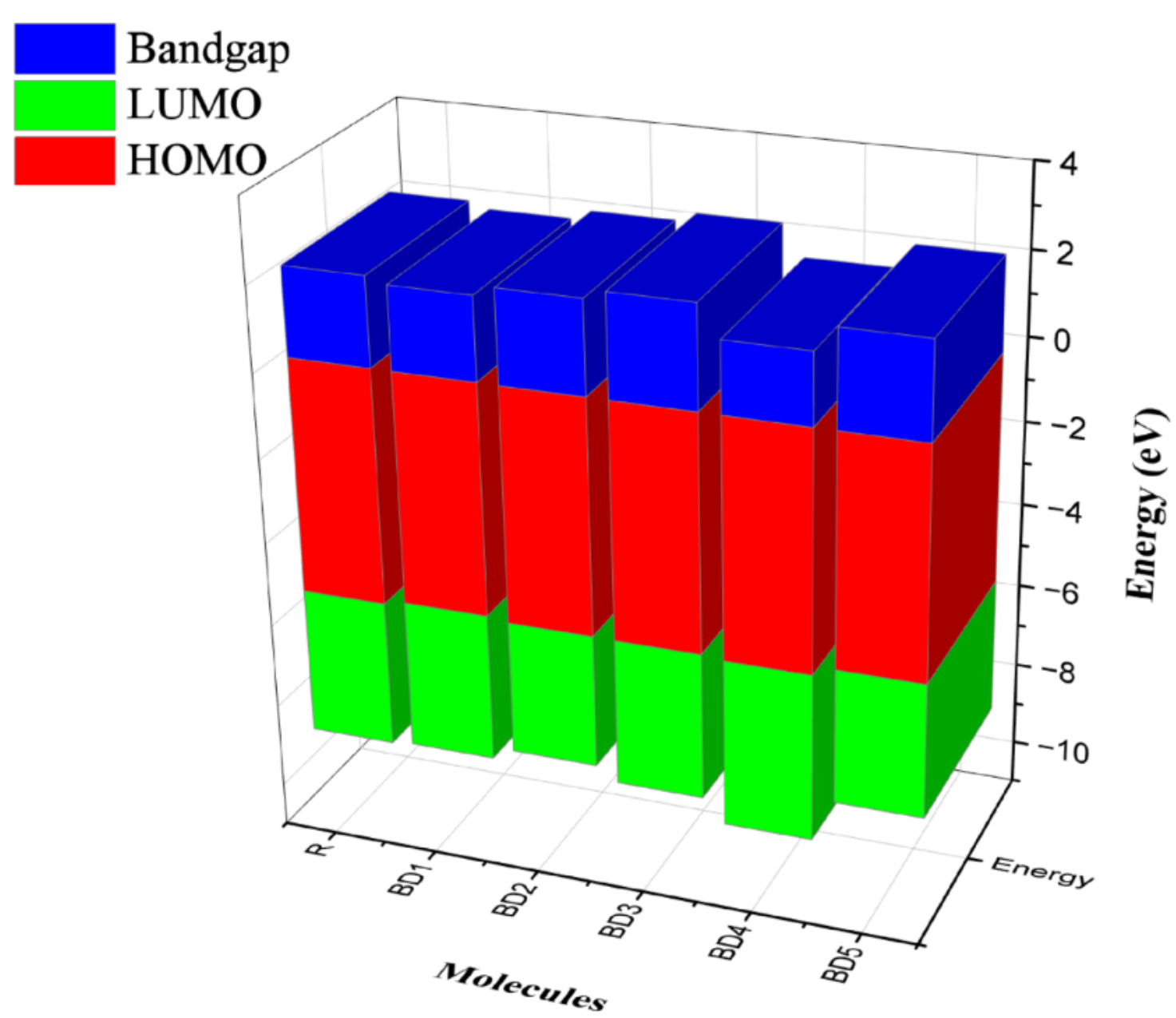

Fig. S3: HOMO-LUMO energies and Bandgap of R and BD1-BD5

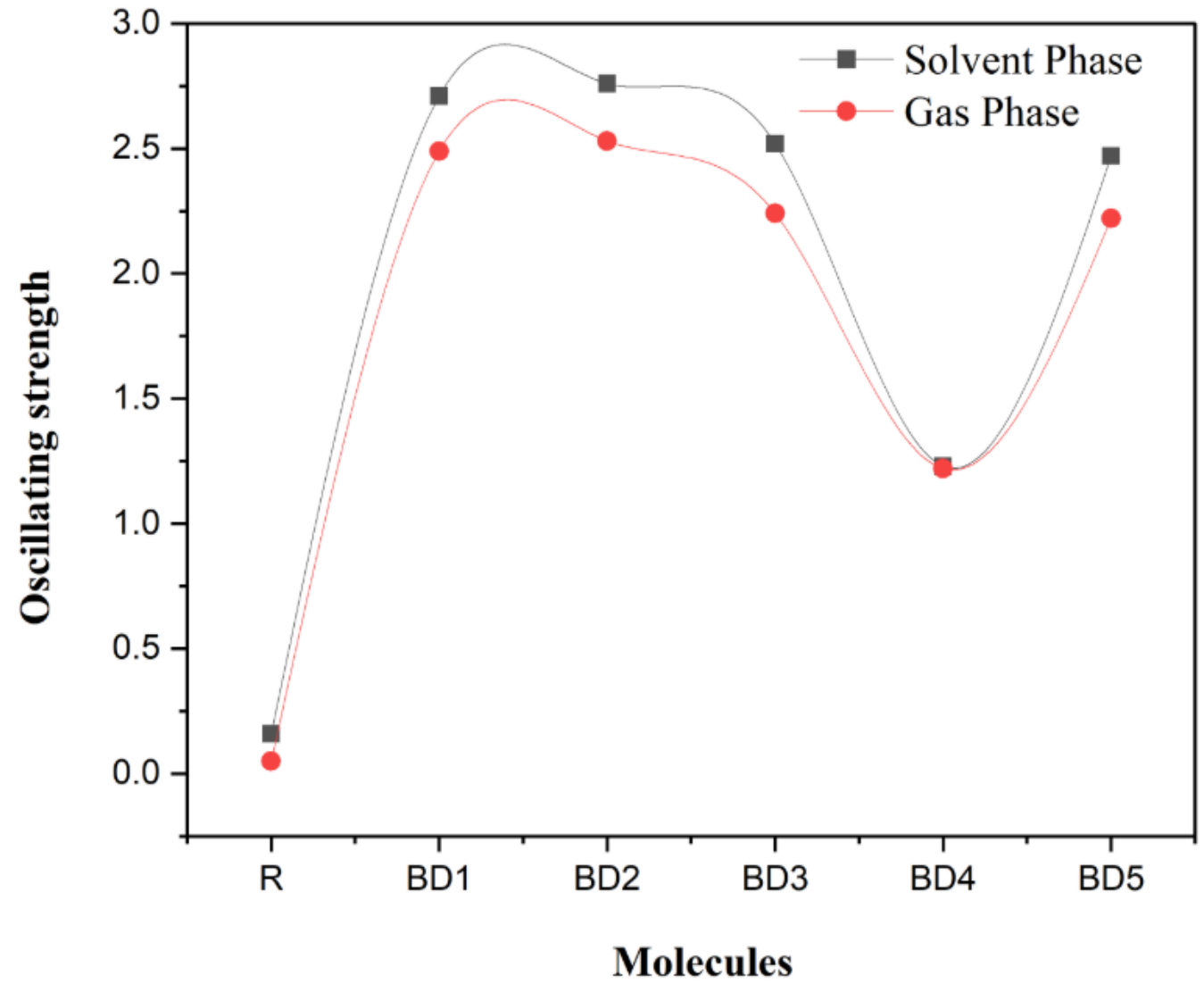

Fig. S4: Representation of the oscillating strength in both phases

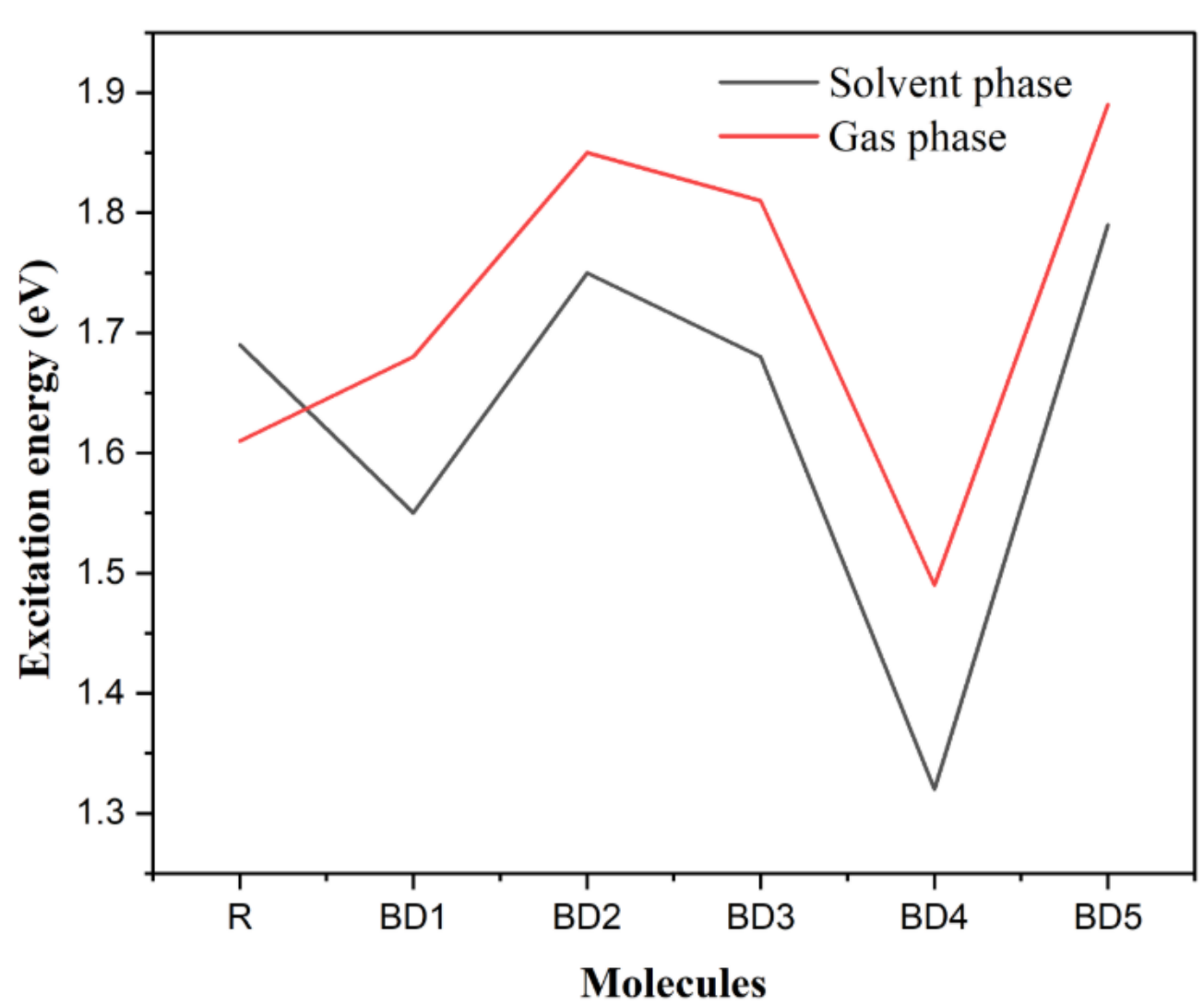

Fig. S5: Illustration of the excitation energy in both phases

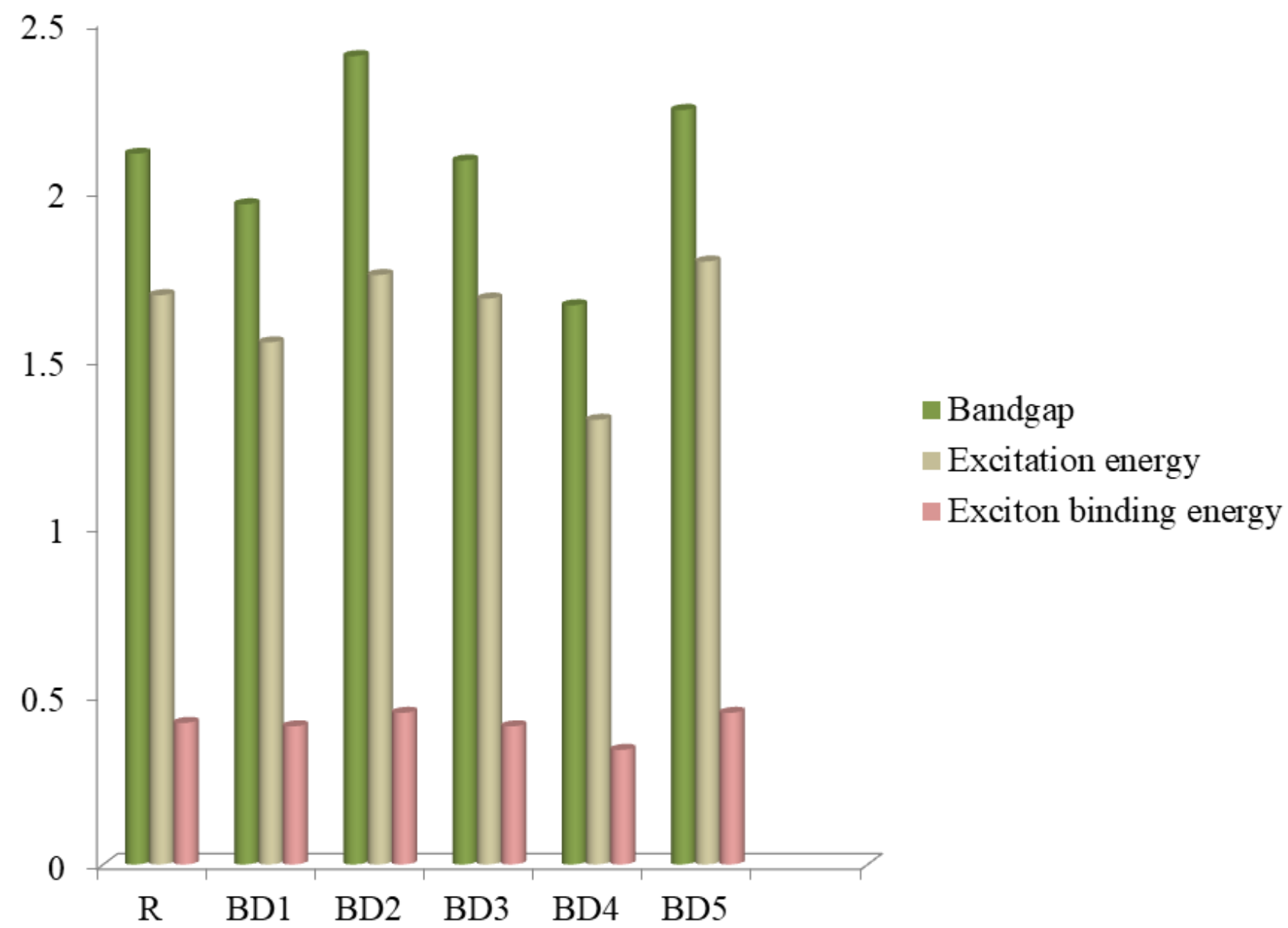

Figure S6: Comparison between Ex, Eopt, and Eg of the modified molecule in the solvent phase

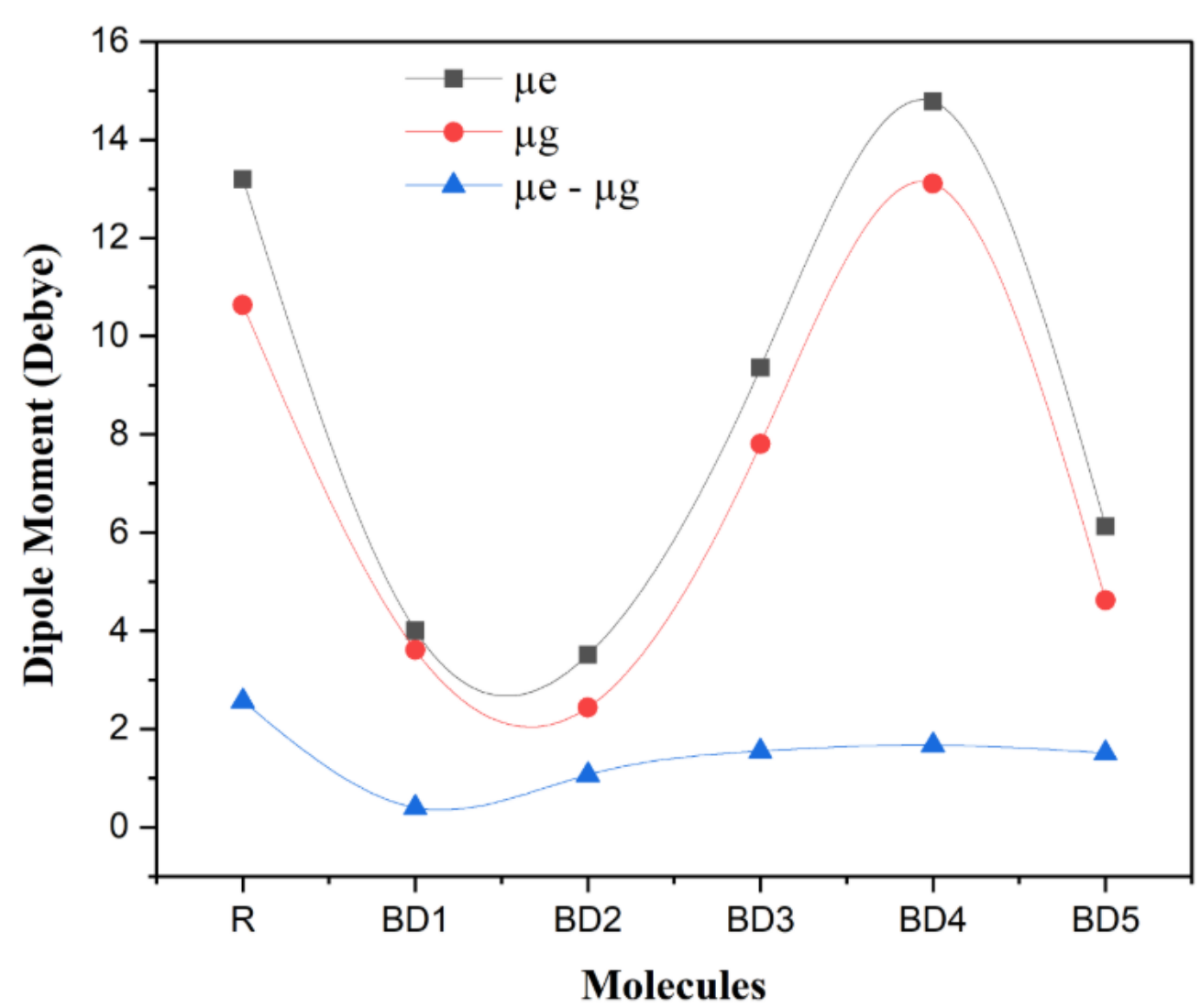

Fig. S7: Representation of dipole moment for both the excited and ground states

Reference Molecule (BZ-E31)

C	0.28075200	0.15382500	-1.85682000
C	-1.15656100	0.25477300	-1.90383100
C	-1.74633000	1.53141300	-2.14890500
C	-0.93861800	2.68331400	-2.30362200
C	0.45053200	2.59531100	-2.13731500
C	1.05873300	1.34101400	-1.90005200
N	0.96775400	3.82491200	-2.23273600
N	-1.26390400	3.96502800	-2.49858600
N	-0.09852500	4.57859100	-2.44080400
C	0.01168100	6.00873800	-2.68592000
C	2.39581000	0.97213200	-1.65920500
C	2.41975500	-0.39613400	-1.47421500
N	1.15409700	-0.92301900	-1.62170100
C	4.65169800	0.16776500	-1.10423500
C	3.71884300	-0.86009800	-1.16502000
S	3.93648800	1.72336700	-1.43111200
C	6.00175300	-0.29778000	-0.93706600
C	6.03636800	-1.69279700	-0.85575800
S	4.47127200	-2.39742500	-0.93531400
C	-3.37656300	0.07067200	-1.63286700

C	-3.12846600	1.39081200	-1.99149600
N	-2.19116000	-0.63453600	-1.61117800
C	-4.72148200	-0.14211200	-1.27482200
C	-5.47297400	1.03477600	-1.41185300
S	-4.53091200	2.40783500	-1.96172200
C	-7.10551800	-0.39579800	-0.60717300
C	-6.81234600	0.91384800	-1.05639700
S	-5.67285600	-1.43543600	-0.65800100
C	-8.40013100	-0.75618800	-0.20125700
C	-8.97939900	-1.91311300	0.28301900
C	-8.28534000	-3.18250800	0.54302000
C	-10.37751700	-2.08839600	0.64149400
O	-7.11317600	-3.44706200	0.35943500
C	-11.41498700	-1.18701000	0.53909500
C	-12.74962100	-1.48892900	0.92143100
N	-13.84549300	-1.69668400	1.22188100
C	-11.27727900	0.13455400	0.03865100
N	-11.20958200	1.21645700	-0.36213600
C	-11.44811900	-5.47589800	2.04141000
C	-11.63646100	-4.16688100	1.63780000
C	-10.53256200	-3.47102400	1.14940400
C	-9.29411600	-4.11276700	1.08660900

C	-9.10316500	-5.42127500	1.48822000
C	-10.20295900	-6.10130200	1.97029500
H	-12.62772900	-3.74673700	1.72094000
H	-8.13291300	-5.89750100	1.43030900
H	-9.07527400	0.08392100	-0.29974000
C	7.25773500	0.39512000	-0.98746000
C	8.92037200	2.29592900	-0.94732100
C	7.62182500	1.67169200	-0.67616300
C	6.83138200	2.62067900	0.14645700
H	8.05097500	-0.24056400	-1.35900800
O	5.75283500	2.43395200	0.66123200
C	8.29083700	5.99247900	1.00101000
C	9.51656200	5.82968300	0.34929300
C	9.83863200	4.66890500	-0.32542200
C	8.89182300	3.64431300	-0.34859800
C	7.66773100	3.82784100	0.29975400
C	7.34967300	4.98492100	0.98768100
H	10.80685800	4.60757600	-0.79972500
H	6.40174100	5.09993900	1.49709400
C	9.98297200	1.77111100	-1.63999200
C	11.19285900	2.48385900	-1.86442100
N	12.18792300	3.03363700	-2.06559300

C	10.00403600	0.46151400	-2.19392000
N	10.06460600	-0.60084200	-2.64327700
C	-2.35075900	-3.04660100	-2.38829200
C	1.03358600	-2.29780000	-2.09923700
C	1.65452600	-2.56409500	-3.48222200
C	-3.62207100	-2.82593600	-3.21212500
C	-3.71372100	-3.72684400	-4.43802100
H	-4.58538900	-3.46644000	-5.04124500
H	-2.82992500	-3.62449400	-5.07467700
C	1.05805300	-1.64993000	-4.55785600
C	1.85577300	-1.62437200	-5.85621600
H	0.02546600	-1.95920300	-4.76231700
H	0.99817200	-0.62885900	-4.17202200
H	2.87696800	-1.27745100	-5.67796300
H	1.91719100	-2.60729000	-6.32862800
H	1.39801300	-0.94518700	-6.57802100
H	-1.52163000	-2.88978400	-3.08826000
H	-0.01530100	-2.55688600	-2.13393200
H	1.49074800	-2.94856700	-1.34839300
C	1.46921300	-4.05170800	-3.82569600
C	2.44104100	-5.02531400	-3.15589000
H	0.43957000	-4.33693200	-3.57901500

H	1.55492100	-4.17885300	-4.90891300
C	3.84173000	-5.01808000	-3.76148300
H	2.50943900	-4.82359500	-2.08001800
H	2.03293200	-6.03875800	-3.23703000
C	4.78296600	-6.00768000	-3.08792800
H	3.76627600	-5.25366100	-4.82896700
H	4.26961800	-4.01220900	-3.70360900
H	5.77587700	-5.98894200	-3.54225700
H	4.89881700	-5.78218000	-2.02385200
H	4.40269400	-7.02990100	-3.16475500
C	-4.27864600	-6.89833300	0.41918900
C	-3.12963700	-6.38856600	-0.44059600
H	-4.08962100	-7.91209900	0.77871700
H	-5.21457100	-6.91264700	-0.14548500
C	-3.35684300	-4.97102500	-0.95638900
H	-2.19750700	-6.41954100	0.13543500
H	-2.98109200	-7.06240100	-1.29231000
C	-2.21168900	-4.47588100	-1.83758900
H	-4.29776600	-4.94365400	-1.51533400
H	-3.50239100	-4.30520800	-0.09873000
H	-1.27313100	-4.54684100	-1.27252600
H	-2.09588700	-5.16382100	-2.68104400

H	-4.43314000	-6.25709100	1.29091100
C	-2.17885600	-2.04082600	-1.23362200
H	-2.98698100	-2.17091100	-0.51056000
H	-1.26000600	-2.24152400	-0.68094000
H	2.72703500	-2.34425200	-3.42233800
C	-7.25644500	7.14731200	6.24690700
C	-6.36219700	6.01842600	6.76103800
H	-8.26136000	6.74947100	6.07121100
H	-7.36851800	7.89579300	7.04016500
C	-4.95826700	6.46369300	7.15338700
H	-6.84887900	5.56212900	7.62929900
H	-6.29601800	5.22531600	6.00833100
H	-4.39709400	6.84435800	6.29682700
H	-4.99364000	7.25885200	7.90393800
C	-7.62585100	5.47200500	1.98683400
C	-7.83629400	6.27955100	3.26330600
H	-7.24021400	6.13259200	1.20005800
H	-6.84319900	4.72603300	2.16523900
C	-6.56518300	6.96156200	3.75923800
H	-8.60899800	7.03897900	3.08832400
H	-8.23442900	5.61770000	4.04085700
C	-6.76054800	7.85588800	4.98383500

H	-5.80519900	6.20010400	3.96985200
H	-6.15266400	7.57155100	2.94766500
H	-5.81621000	8.36591200	5.20057200
H	-7.47357900	8.64924300	4.73119800
C	-7.76870200	2.06576400	-1.09031900
C	-7.82892700	2.82501000	0.24344400
H	-8.77391900	1.73983900	-1.36773400
H	-7.44803100	2.75399000	-1.87881700
C	-8.74917300	4.03906200	0.16884400
H	-8.18283200	2.14628800	1.02699400
H	-6.81596600	3.12477000	0.52740500
C	-8.89772900	4.79086200	1.49047400
H	-8.37676800	4.72841700	-0.59934900
H	-9.73654400	3.70516200	-0.16439500
H	-9.67946700	5.54937500	1.37195200
H	-9.26569800	4.09970800	2.25875000
H	-4.38514300	5.63516100	7.57583700
C	9.46270300	-3.50449300	2.42848700
C	8.93302500	-3.49888400	0.99885500
H	8.65835200	-3.80097800	3.11265900
H	9.74248400	-2.48343000	2.71475300
C	7.73351800	-2.57855200	0.80931100

H	9.73422500	-3.19609100	0.31438100
H	8.65680900	-4.52045600	0.71053300
C	7.21960800	-2.57784900	-0.63719000
H	6.92075400	-2.88048900	1.47825700
H	8.00045600	-1.55605300	1.09545900
H	8.02872200	-2.27234100	-1.31041200
H	6.96052200	-3.59952900	-0.92944000
C	13.01761200	-5.31000600	5.63708600
C	12.38804600	-5.36252700	4.24428400
H	13.90270600	-5.95651100	5.64569000
H	13.38581800	-4.29346700	5.81766000
C	11.19740300	-4.42852700	4.05277700
H	13.15837100	-5.10674500	3.50787300
H	12.08401600	-6.38892400	4.00943900
C	10.65923500	-4.42832600	2.62615700
H	10.38910600	-4.70747100	4.73822700
H	11.49066400	-3.40805100	4.32981600
H	11.46040400	-4.13591700	1.93663800
H	10.37751000	-5.45067000	2.34540100
C	10.77267700	-7.54095200	7.95315600
C	11.59665900	-7.15085200	6.73276500
H	10.41864500	-8.57217400	7.88639700

H	11.35996100	-7.45047300	8.87110000
H	9.89599800	-6.89591300	8.06002500
C	12.10500300	-5.71384800	6.79576500
H	12.45179500	-7.82993600	6.63467200
H	10.99310900	-7.29320000	5.83019400
H	11.24982000	-5.03072000	6.85582900
H	12.65666000	-5.57762000	7.73303900
C	1.11068400	6.62652400	-1.84096200
C	0.71258400	6.64499700	-0.37250700
O	-0.38168400	6.99462900	-0.03198700
O	1.61325300	6.30183000	0.55968400
C	2.87851400	5.74676500	0.21469600
H	3.32820400	5.45005200	1.15928400
H	2.76707500	4.86614000	-0.41880800
H	3.52025300	6.48690100	-0.26999000
H	0.21050100	6.16521300	-3.74875800
H	-0.95562400	6.43626000	-2.43250600
H	2.05509700	6.12572700	-2.03618600
H	1.21948000	7.67238100	-2.14210300
H	-4.50714200	-2.96972300	-2.58817000
H	-3.64873900	-1.78332500	-3.54157400
H	-3.80865100	-4.78088000	-4.16787900

F	10.38688500	6.83089900	0.39378600
F	8.05517000	7.13596100	1.63156900
F	-12.47580300	-6.17385300	2.51456700
F	-10.09920700	-7.36283200	2.37648200

1 2 1.5 6 1.5 13 1.0

2 3 1.5 22 1.0

3 4 1.5 21 1.5

4 5 1.5 8 1.5

5 6 1.5 7 1.5

6 11 1.5

7 9 1.0

8 9 1.0

9 10 1.0

10 182 1.0 190 1.0 191 1.0

11 12 2.0 16 1.0

12 13 1.5 15 1.5

13 68 1.0

14 15 1.5 16 1.0 17 1.5

15 19 1.0

16

17 18 1.5 48 1.5

18 19 1.0 155 1.0

19

20 21 1.5 22 1.5 23 1.5

21 25 1.0

22 110 1.0

23 24 1.5 28 1.0

24 25 1.0 27 1.5

25

26 27 1.5 28 1.0 29 1.5

27 135 1.0

28

29 30 2.0 47 1.0

30 31 1.0 32 1.0

31 33 2.0 42 1.0

32 34 2.0 41 1.0

33

34 35 1.5 37 1.5

35 36 3.0

36

37 38 3.0

38

39 40 2.0 44 1.5 199 1.0

40 41 1.5 45 1.0

41 42 1.5

42 43 2.0

43 44 2.0 46 1.0

44 200 1.0

45

46

47

48 50 2.0 52 1.0

49 50 1.0 57 1.0 62 2.0

50 51 1.0

51 53 2.0 58 1.0

52

53

54 55 1.5 59 2.0 198 1.0

55 56 2.0 197 1.0

56 57 1.5 60 1.0

57 58 1.5

58 59 2.0

59 61 1.0

60

61

62 63 1.5 65 1.5

63 64 3.0

64

65 66 3.0

66

67 70 1.0 81 1.0 104 1.0 110 1.0

68 69 1.0 82 1.0 83 1.0

69 74 1.0 84 1.0 113 1.0

70 71 1.0 194 1.0 195 1.0

71 72 1.0 73 1.0 196 1.0

72

73

74 75 1.0 76 1.0 77 1.0

75 78 1.0 79 1.0 80 1.0

76

77

78

79

80

81

82

83

84 85 1.0 86 1.0 87 1.0

85 88 1.0 89 1.0 90 1.0

86

87

88 91 1.0 92 1.0 93 1.0

89

90

91 94 1.0 95 1.0 96 1.0

92

93

94

95

96

97 98 1.0 99 1.0 100 1.0 109 1.0

98 101 1.0 102 1.0 103 1.0

99

100

101 104 1.0 105 1.0 106 1.0

102

103

104 107 1.0 108 1.0

105

106

107

108

109

110 111 1.0 112 1.0

111

112

113

114 115 1.0 116 1.0 117 1.0 130 1.0

115 118 1.0 119 1.0 120 1.0

116

117

118 121 1.0 122 1.0 147 1.0

119

120

121

122

123 124 1.0 125 1.0 126 1.0 142 1.0

124 127 1.0 128 1.0 129 1.0

125

126

127 130 1.0 131 1.0 132 1.0

128

129

130 133 1.0 134 1.0

131

132

133

134

135 136 1.0 137 1.0 138 1.0

136 139 1.0 140 1.0 141 1.0

137

138

139 142 1.0 143 1.0 144 1.0

140

141

142 145 1.0 146 1.0

143

144

145

146

147

148 149 1.0 150 1.0 151 1.0 167 1.0

149 152 1.0 153 1.0 154 1.0

150

151

152 155 1.0 156 1.0 157 1.0

153

154

155 158 1.0 159 1.0

156

157

158

159

160 161 1.0 162 1.0 163 1.0 177 1.0

161 164 1.0 165 1.0 166 1.0

162

163

164 167 1.0 168 1.0 169 1.0

165

166

167 170 1.0 171 1.0

168

169

170

171

172 173 1.0 174 1.0 175 1.0 176 1.0

173 177 1.0 178 1.0 179 1.0

174

175

176

177 180 1.0 181 1.0

178

179

180

181

182 183 1.0 192 1.0 193 1.0

183 184 2.0 185 1.5

184

185 186 1.0

186 187 1.0 188 1.0 189 1.0

187

188

189

190

191

192

193

194

195

196

197

198

199

200

BD1

C	0.68714900	2.29598600	-0.17397900
C	-0.73395200	2.32813300	-0.20581100
C	-1.41507300	3.56301700	-0.34019200
C	-0.66495600	4.76657800	-0.31176600
C	0.73199100	4.73719000	-0.12422800
C	1.42572300	3.50130600	-0.06653200
N	1.17545700	5.99705300	-0.07808900
N	-1.05483900	6.04293600	-0.37472900
N	0.07168200	6.70701000	-0.22365700
C	0.09906800	8.15891300	-0.30605500
C	2.78116500	3.13045200	-0.01372700
C	2.84334900	1.74454900	-0.12220500
N	1.57371000	1.22661300	-0.23132600

C	5.09173200	2.30337000	-0.02145000
C	4.16420200	1.26300800	-0.13590700
S	4.34240100	3.87904700	0.09400200
C	6.42271300	1.87266600	-0.02620000
C	6.53261800	0.49885800	-0.14109900
S	4.95241300	-0.26699000	-0.22687800
C	-2.91305400	1.87728200	-0.24913900
C	-2.78691000	3.25607000	-0.38799400
N	-1.66967400	1.30191400	-0.12718400
C	-4.25557100	1.45989400	-0.21341800
C	-5.13122100	2.53920800	-0.35320700
S	-4.31060200	4.07512200	-0.50695600
C	-6.65855500	0.80934600	-0.21844000
C	-6.48432100	2.17269400	-0.35423000
S	-5.11739400	-0.02962100	-0.10206600
C	1.33002700	-0.08013000	-0.81635500
H	1.27638500	-0.86668700	-0.06130200
C	-1.49011000	0.00257100	0.49617900
H	-1.47167500	-0.80741000	-0.23549700
H	-2.30980700	-0.17368300	1.19352700
C	1.17730600	8.74454700	0.58193500
C	0.82026200	8.61476100	2.05059800

O	-0.27867700	8.32838100	2.42816800
O	1.78182100	8.87056600	2.95472700
C	3.11433500	9.16243900	2.55662600
H	3.64646500	9.39925300	3.47473900
H	3.58830400	8.29893800	2.08447200
H	3.16218700	10.02523100	1.88831700
H	0.27108700	8.44107700	-1.34756600
H	-0.88896200	8.49367300	0.00035500
H	2.13436800	8.27078300	0.36427400
H	1.28645000	9.80846900	0.34766800
H	0.40290200	-0.05977600	-1.38457300
H	2.13675800	-0.31535100	-1.51138600
H	-0.56524500	-0.00477500	1.06856000
C	10.14811400	-1.06409400	0.07816500
C	9.28241800	-2.02246000	-0.46077300
C	7.94217400	-1.58164200	-0.62629000
C	7.73165400	-0.27433300	-0.16898400
S	9.20777800	0.38161300	0.42207500
C	-9.64541000	-1.72352900	0.28398900
C	-10.14129400	-0.56685500	-0.32992500
C	-9.16647800	0.44689900	-0.55070200
C	-7.88230000	0.07454400	-0.13194400

S	-7.92749100	-1.49097200	0.57162700
C	-11.07548100	1.52440200	-1.24508000
C	-9.72803000	1.62716800	-1.10947100
S	-11.71872500	-0.03198900	-0.77404200
C	7.83645000	-3.63784800	-1.67717600
C	7.14089000	-2.52230200	-1.32877100
S	9.51144700	-3.58574700	-1.14585500
H	6.10844700	-2.39174300	-1.61512000
C	7.20439500	-4.70125100	-2.50612900
C	-11.92768300	2.59348400	-1.78937000
C	-14.11926100	3.20196000	-2.34001200
C	8.47934700	-6.47826000	-1.44042500
O	7.68678900	-5.95291500	-2.50191100
O	-13.21308000	2.22554500	-1.82483000
H	8.31849700	-7.55398500	-1.46520200
H	9.53970500	-6.27614400	-1.59977200
H	8.16808500	-6.08932800	-0.47036500
H	-13.85943200	3.45598500	-3.36773400
H	-15.10195600	2.74105000	-2.29591600
H	-14.09040400	4.10557200	-1.73095000
H	7.28238200	2.52646900	0.02535700
O	6.25979000	-4.43896400	-3.19904200

O	-11.51603100	3.66493800	-2.15163800
C	11.54132900	-0.93002500	0.24151700
C	-10.19753600	-2.88175700	0.86843800
C	12.33126600	-3.27976800	0.74169100
C	12.53741700	-1.87283600	0.35197500
C	13.97201200	-1.62248400	0.32099100
C	-11.96660800	-4.58889900	1.46167900
C	-12.21525300	-3.38818000	-0.56689600
C	-11.39747200	-3.51915800	0.65432500
H	11.85998700	0.10535400	0.28386300
H	-9.54848400	-3.32717700	1.61410000
C	13.99600900	-5.15081800	1.31193400
C	13.67565500	-3.86097900	0.93417200
C	14.64830900	-2.88443700	0.69324200
C	15.99416700	-3.21689600	0.82950000
C	16.32109000	-4.51702200	1.20411300
C	15.34053800	-5.47754700	1.44355900
H	13.20836300	-5.87131400	1.49523900
H	16.78264200	-2.49960800	0.65860400
H	17.36545700	-4.78314500	1.31329600
H	15.63111100	-6.47982600	1.73424900
C	-13.14696200	-5.10872600	0.73707800

C	-14.05607000	-6.12017500	1.03910300
C	-15.08220700	-6.38029100	0.13539600
C	-15.21334500	-5.65904100	-1.04962100
C	-14.30727500	-4.65119300	-1.35809800
C	-13.29388200	-4.39197200	-0.45547800
H	-13.98449800	-6.70477200	1.94384300
H	-15.79294400	-7.16564600	0.36252100
H	-16.02445500	-5.88836000	-1.73012000
H	-14.37687100	-4.07372500	-2.27176500
C	-11.54477900	-5.02355300	2.69650700
C	14.61398600	-0.45984500	-0.03542900
C	13.93865300	0.72153800	-0.44767300
C	16.02853100	-0.31953600	-0.04004500
C	-10.44143400	-4.46329300	3.39665300
C	-12.17720400	-6.07712200	3.41096800
O	11.27365200	-3.84104400	0.94962900
O	-12.00282400	-2.66969100	-1.52204000
N	17.17281200	-0.16418300	-0.05451400
N	13.42471300	1.69955400	-0.78559400
N	-9.55030100	-4.03529800	3.99468700
N	-12.66062400	-6.92973600	4.02188500
H	-7.30455900	2.87202500	-0.40346700

H -9.19772000 2.51814300 -1.40822700

1 2 1.5 6 1.5 13 1.0

2 3 1.5 22 1.0

3 4 1.5 21 1.5

4 5 1.5 8 1.5

5 6 1.5 7 1.5

6 11 1.5

7 9 1.0

8 9 1.0

9 10 1.0

10 34 1.0 42 1.0 43 1.0

11 12 1.5 16 1.0

12 13 1.5 15 1.5

13 29 1.0

14 15 1.5 16 1.0 17 1.5

15 19 1.0

16

17 18 2.0 78 1.0

18 19 1.0 52 1.5

19

20 21 1.5 22 1.5 23 1.5

21 25 1.0

22 31 1.0

23 24 1.5 28 1.0

24 25 1.0 27 1.5

25

26 27 2.0 28 1.0 57 1.5

27 123 1.0

28

29 30 1.0 46 1.0 47 1.0

30

31 32 1.0 33 1.0 48 1.0

32

33

34 35 1.0 44 1.0 45 1.0

35 36 2.0 37 1.0

36

37 38 1.0

38 39 1.0 40 1.0 41 1.0

39

40

41

42

43

44

45

46

47

48

49 50 1.5 53 1.0 81 1.5

50 51 1.5 64 1.0

51 52 1.5 63 1.5

52 53 1.0

53

54 55 1.5 58 1.0 82 1.5

55 56 1.5 61 1.0

56 57 1.5 60 1.5

57 58 1.0

58

59 60 2.0 61 1.0 67 1.0

60 124 1.0

61

62 63 2.0 64 1.0 66 1.0

63 65 1.0

64

65

66 70 1.5 79 2.0

67 71 1.5 80 2.0

68 71 1.0 75 1.0 76 1.0 77 1.0

69 70 1.0 72 1.0 73 1.0 74 1.0

70

71

72

73

74

75

76

77

78

79

80

81 84 2.0 89 1.0

82 88 2.0 90 1.0

83 84 1.0 92 1.0 117 2.0

84 85 1.0

85 93 1.0 112 2.0

86 88 1.0 101 1.0 111 2.0

87 88 1.0 106 1.0 118 2.0

88

89

90

91 92 2.0 96 1.5 97 1.0

92 93 1.5

93 94 1.5

94 95 1.5 98 1.0

95 96 1.5 99 1.0

96 100 1.0

97

98

99

100

101 102 1.5 106 1.5

102 103 1.5 107 1.0

103 104 1.5 108 1.0

104 105 1.5 109 1.0

105 106 2.0 110 1.0

106

107

108

109

110

111 115 1.5 116 1.5

112 113 1.5 114 1.5

113 120 3.0

114 119 3.0

115 121 3.0

116 122 3.0

117

118

119

120

121

122

123

124

BD2

C	0.71389200	2.07861300	-0.19384600
C	-0.71741900	2.08048800	-0.14790800
C	-1.41343800	3.31759200	-0.10037700
C	-0.69852500	4.53502500	-0.20356100
C	0.69608700	4.52747500	-0.37500900

C	1.41117900	3.30595400	-0.35919800
N	1.11498300	5.79295500	-0.48191000
N	-1.11904600	5.80407800	-0.20484900
N	-0.00374700	6.48545200	-0.36478700
C	-0.02709600	7.93333500	-0.49939600
C	2.77935100	2.99503300	-0.41926100
C	2.90302100	1.62218100	-0.26271300
N	1.65819400	1.04983200	-0.12532700
C	5.12317500	2.27641000	-0.43931800
C	4.24882500	1.20179200	-0.26455700
S	4.29990500	3.81056600	-0.59752000
C	6.47324800	1.90448400	-0.49608700
C	6.65181400	0.54275100	-0.35727300
S	5.11498800	-0.28599800	-0.15451700
C	-2.90629200	1.63489800	-0.02785100
C	-2.78220200	3.01635700	-0.00646600
N	-1.66262600	1.04956600	-0.11214600
C	-4.25274900	1.21801500	0.02580700
C	-5.12352000	2.30571600	0.09100600
S	-4.30052100	3.84745000	0.09403300
C	-6.65923700	0.57973500	0.16027800
C	-6.47898000	1.94602200	0.17140200

S	-5.12493600	-0.27154800	0.05314100
C	1.52361800	-0.27276900	0.47686900
C	-1.53379900	-0.32218600	-0.59363000
H	-2.53351700	-0.75364400	-0.54697900
C	1.24026000	8.55921100	0.04481000
C	1.28901400	8.49135200	1.55967500
O	0.32634200	8.25338400	2.22963300
O	2.46229100	8.75216000	2.16278600
C	3.64716400	8.97774500	1.41160700
H	4.41039400	9.23243900	2.14301700
H	3.95612800	8.07820600	0.87450200
H	3.53645000	9.80993400	0.71256700
H	-0.14720100	8.17889700	-1.55739200
H	-0.90139200	8.27176300	0.05133600
H	2.11031200	8.08092800	-0.40375100
H	1.26639600	9.61380300	-0.24879600
H	0.92304500	-0.91333000	-0.16632400
H	2.52166600	-0.71065000	0.47090800
H	-0.93383900	-0.90538000	0.10278200
C	10.34955000	-0.81214800	-0.12013100
C	9.51048900	-1.88142400	-0.43069800
C	8.13752900	-1.53113700	-0.57467800

C	7.88728400	-0.17730600	-0.33367900
S	9.36172600	0.62892500	0.03583900
C	-9.65438900	-1.95173900	0.63195700
C	-10.16041500	-0.76314200	0.10497000
C	-9.17681200	0.24986600	-0.10311600
C	-7.88648800	-0.15584100	0.24657700
S	-7.92771700	-1.75665500	0.86580500
C	-11.08963300	1.38221200	-0.68742700
C	-9.73780900	1.46173200	-0.59104300
S	-11.74865400	-0.18299900	-0.26218500
C	8.07491300	-3.74517700	-1.23154300
C	7.34339300	-2.61581100	-1.03985900
S	9.77802300	-3.53643600	-0.84784500
H	6.28880700	-2.57955900	-1.26782900
C	7.46126900	-4.97237200	-1.80751600
C	-11.94035400	2.48694600	-1.15668600
C	-14.13655200	3.15198900	-1.61369400
C	8.81374000	-6.44734100	-0.42666900
O	7.99934500	-6.17855800	-1.56615900
O	-13.23293400	2.14060400	-1.16893300
H	8.68153400	-7.50756800	-0.22057600
H	9.86685000	-6.25446200	-0.63546500

H	8.49807300	-5.86842000	0.44183700
H	-13.90282600	3.44755700	-2.63668400
H	-15.12528100	2.70487800	-1.56086600
H	-14.07544700	4.02790700	-0.96744900
H	7.29489500	2.58951300	-0.65297800
O	6.48846500	-4.89197200	-2.50633400
O	-11.52499900	3.56865000	-1.48399300
C	11.74750100	-0.61016800	-0.02027300
C	-10.19497900	-3.16546400	1.12562100
C	12.86201100	-2.85329800	0.62294700
C	12.79662300	-1.45028400	0.18978000
C	14.18249000	-0.92113000	0.11913600
C	-11.68839900	-5.02433900	1.72783200
C	-12.46451500	-3.59787100	-0.03228700
C	-11.38266100	-3.80056800	0.94179800
H	12.06567600	0.42590800	-0.14433300
H	-9.53857400	-3.70871800	1.80722400
C	14.91166500	-4.38008100	1.09119800
C	14.30928100	-3.18431600	0.73299600
C	15.08090800	-2.06303700	0.44126400
C	16.46366400	-2.10436700	0.48954900
C	17.08950300	-3.30182700	0.83680500

C	16.29836700	-4.42342400	1.13468400
H	14.31029400	-5.24891300	1.32955100
H	17.04137200	-1.21526500	0.26385100
H	16.79278500	-5.34903300	1.41021800
C	-13.01758600	-5.49562700	1.26122300
C	-13.77409400	-6.57882800	1.68012400
C	-14.99111700	-6.80391600	1.05204300
C	-15.46052000	-5.97617500	0.01892600
C	-14.68010800	-4.89576700	-0.39291400
C	-13.47034600	-4.66562800	0.23957600
H	-13.41579100	-7.22679300	2.47072800
H	-15.60136500	-7.64616500	1.36073400
H	-15.00599100	-4.24186700	-1.19387400
O	11.94382900	-3.59860200	0.90570700
O	-12.52548300	-2.78644800	-0.93353700
H	-7.29236700	2.64749000	0.27899700
H	-9.19914800	2.35532200	-0.86716400
C	-1.01022700	-0.41653300	-2.01792800
C	0.99627100	-0.23625100	1.90237400
H	-0.00853400	0.00602300	-2.11195300
H	-0.97026300	-1.46214200	-2.33033100
H	-1.66854400	0.11986900	-2.70297500

H	1.65439800	0.35759500	2.53845600
H	-0.00398100	0.19665400	1.95466300
H	0.95182500	-1.24946000	2.30730500
O	-10.99241400	-5.53696300	2.57630800
O	14.51783800	0.21225000	-0.14386000
C	-16.79345800	-6.25014600	-0.61492800
C	18.58635300	-3.40606600	0.87956500
H	-16.90906100	-5.70212000	-1.55024400
H	-16.92206400	-7.31420600	-0.82377000
H	-17.60778700	-5.94851500	0.05054200
H	19.05374700	-2.42121700	0.87636300
H	18.92152800	-3.94088400	1.77062500
H	18.96085000	-3.95496700	0.01045800

1 2 1.5 6 1.5 13 1.0

2 3 1.5 22 1.0

3 4 1.5 21 1.5

4 5 1.5 8 1.5

5 6 1.5 7 1.5

6 11 1.5

7 9 1.0

8 9 1.0

9 10 1.0

10 32 1.0 40 1.0 41 1.0

11 12 1.5 16 1.0

12 13 1.5 15 1.5

13 29 1.0

14 15 1.5 16 1.0 17 1.5

15 19 1.0

16

17 18 2.0 76 1.0

18 19 1.0 50 1.5

19

20 21 1.5 22 1.5 23 1.5

21 25 1.0

22 30 1.0

23 24 1.5 28 1.0

24 25 1.0 27 1.5

25

26 27 2.0 28 1.0 55 1.5

27 109 1.0

28

29 44 1.0 45 1.0 112 1.0

30 31 1.0 46 1.0 111 1.0

31

32 33 1.0 42 1.0 43 1.0

33 34 2.0 35 1.0

34

35 36 1.0

36 37 1.0 38 1.0 39 1.0

37

38

39

40

41

42

43

44

45

46

47 48 1.5 51 1.0 79 1.5

48 49 1.5 62 1.0

49 50 1.5 61 1.5

50 51 1.0

51

52 53 1.5 56 1.0 80 1.5

53 54 1.5 59 1.0

54 55 1.5 58 1.5

55 56 1.0

56

57 58 2.0 59 1.0 65 1.0

58 110 1.0

59

60 61 2.0 62 1.0 64 1.0

61 63 1.0

62

63

64 68 1.5 77 2.0

65 69 1.5 78 2.0

66 69 1.0 73 1.0 74 1.0 75 1.0

67 68 1.0 70 1.0 71 1.0 72 1.0

68

69

70

71

72

73

74

75

76

77

78

79 82 2.0 87 1.0

80 86 2.0 88 1.0

81 82 1.0 90 1.0 107 2.0

82 83 1.0

83 91 1.0 120 2.0

84 86 1.0 98 1.0 119 2.0

85 86 1.0 103 1.0 108 2.0

86

87

88

89 90 1.5 94 1.5 95 1.0

90 91 1.5

91 92 2.0

92 93 1.5 96 1.0

93 94 1.5 122 1.0

94 97 1.0

95

96

97

98 99 1.5 103 1.5

99 100 1.5 104 1.0

100 101 1.5 105 1.0

101 102 1.5 121 1.0

102 103 2.0 106 1.0

103

104

105

106

107

108

109

110

111 113 1.0 114 1.0 115 1.0

112 116 1.0 117 1.0 118 1.0

113

114

115

116

117

118

119

120

121 123 1.0 124 1.0 125 1.0

122 126 1.0 127 1.0 128 1.0

123

124

125

126

127

128

BD3

C	0.72220100	1.85950300	0.08012300
C	-0.70146500	1.87666500	0.02704100
C	-1.38407000	3.08341800	-0.25400000
C	-0.65198800	4.29436500	-0.32627100
C	0.73852000	4.29975700	-0.10235800
C	1.44063000	3.08168900	0.07752700
N	1.16984900	5.56311800	-0.17188100
N	-1.05254500	5.55345200	-0.52725600
N	0.06328600	6.24175700	-0.41523000
C	0.07863700	7.67956100	-0.63496000
C	2.80150900	2.74130000	0.13955100
C	2.89443800	1.35208100	0.13809500
N	1.62860800	0.80118500	0.10890900
C	5.12927600	1.98938300	0.08430100
C	4.23317700	0.91346400	0.08852900
S	4.33609500	3.54454400	0.12651600

C	6.47326200	1.60988100	0.02776800
C	6.63298700	0.23703800	-0.01815300
S	5.08194700	-0.58685800	0.02647900
C	-2.87329400	1.39657700	-0.08858100
C	-2.75174900	2.75568700	-0.34500500
N	-1.63192500	0.83824700	0.14965500
C	-4.21348900	0.96941900	-0.09304400
C	-5.08623100	2.01831100	-0.39331800
S	-4.26636900	3.54092600	-0.64626400
C	-6.60740500	0.29271600	-0.17598500
C	-6.43459200	1.63715500	-0.43898900
S	-5.07086000	-0.51048200	0.11957600
C	1.39807600	-0.54710300	-0.41251700
C	-1.53397100	-0.23176300	1.14642800
H	-2.19531100	-1.03946200	0.82346400
C	1.11950700	8.36214000	0.22806700
C	0.71858800	8.35870500	1.69103400
O	-0.38320300	8.07350100	2.06113700
O	1.64296200	8.72680100	2.59512800
C	2.97776500	9.02565500	2.21019600
H	3.47296700	9.36351200	3.11725900
H	3.49301400	8.13898900	1.83390300

H	3.02007100	9.82459100	1.46642200
H	0.28335100	7.86338300	-1.69238400
H	-0.92300700	8.02788000	-0.39521700
H	2.08996000	7.88705600	0.08438700
H	1.22101100	9.40178200	-0.09973000
H	1.99276200	-0.66054300	-1.32399900
H	-0.52579200	-0.62936400	1.12503100
C	10.31914700	-1.17585800	0.06607300
C	9.45312000	-2.20802000	-0.32042300
C	8.08887400	-1.82823500	-0.42543300
C	7.85864000	-0.49072900	-0.07274300
S	9.34494900	0.26770500	0.33928500
C	-9.61585600	-2.20605400	0.39110300
C	-10.06650100	-1.13161000	-0.38920400
C	-9.07996700	-0.13899800	-0.64987700
C	-7.83060700	-0.44414600	-0.09326600
S	-7.92349800	-1.91524500	0.78279100
C	-10.92327700	0.81778600	-1.63242500
C	-9.59277900	0.95529900	-1.39773100
S	-11.60123400	-0.67441000	-1.02220200
C	7.98526400	-3.98392300	-1.25050200
C	7.27367100	-2.86118600	-0.96169600

S	9.69278800	-3.82070300	-0.86681800
H	6.21766100	-2.79617800	-1.17513900
C	7.32982500	-5.14727300	-1.91144000
C	-11.72890700	1.79851100	-2.37831600
C	-13.86587000	2.29566000	-3.19362200
C	8.79218000	-6.76294600	-0.82556300
O	7.86332100	-6.37437400	-1.83471900
O	-13.00330100	1.40821000	-2.48027600
H	8.69198400	-7.84295900	-0.74285400
H	9.81671200	-6.52541600	-1.11600700
H	8.56138200	-6.30499700	0.13677200
H	-13.51510900	2.42354600	-4.21771600
H	-14.84493900	1.82539100	-3.17986900
H	-13.89889400	3.26866200	-2.70322200
H	7.30579800	2.29913200	-0.00391200
O	6.31505200	-4.98670600	-2.53307900
O	-11.29091100	2.82330900	-2.83297000
C	11.70447400	-0.97436900	0.14738900
C	-10.17973500	-3.31423800	1.04313600
C	12.71343200	-3.22787600	0.58260500
C	12.79680800	-1.82006400	0.21834000
C	-12.22320900	-3.93019100	-0.27056100

C	-11.37030500	-4.00315000	0.91079700
H	11.99902000	0.07389400	0.12679700
H	-9.56623200	-3.70805600	1.85234400
O	11.68291800	-3.77839600	0.92437800
O	-11.91894300	-3.32919900	-1.28266400
H	-7.25527500	2.31696700	-0.60881900
H	-9.03932900	1.80738700	-1.76102800
H	-13.16543300	-4.49651700	-0.23839300
H	13.65548200	-3.79497300	0.58611200
C	14.08957900	-1.12926100	0.07932100
C	-11.61223800	-4.98002500	1.98514600
O	-10.87625400	-5.15719600	2.92817000
O	14.21333200	0.05776700	-0.11481100
C	15.39627900	-1.95617200	0.17914600
C	-12.89205200	-5.85178600	1.93022300
F	-13.99594000	-5.08616900	1.89124700
F	-12.89855400	-6.63092400	0.83627300
F	-12.97663400	-6.63565100	2.99063600
F	16.45562300	-1.19296800	-0.02188300
F	15.51670300	-2.52344800	1.39079500
F	15.41483400	-2.94143300	-0.73484600
H	0.35640000	-0.60238000	-0.72449800

C	-1.88077500	0.22800400	2.55477600
C	1.74145600	-1.65485400	0.57159300
H	-1.78541700	-0.60755800	3.25130200
H	-1.20692800	1.02271300	2.87987700
H	-2.90458000	0.60064700	2.61199600
H	2.79920200	-1.65073100	0.83550300
H	1.17909600	-1.56692200	1.50229900
H	1.51839000	-2.62522700	0.12385000

1 2 1.5 6 1.5 13 1.0

2 3 1.5 22 1.0

3 4 1.5 21 1.5

4 5 1.5 8 1.5

5 6 1.5 7 1.5

6 11 1.5

7 9 1.0

8 9 1.5

9 10 1.0

10 32 1.0 40 1.0 41 1.0

11 12 1.5 16 1.0

12 13 1.5 15 1.5

13 29 1.0

14 15 1.5 16 1.0 17 1.5

15 19 1.0

16

17 18 2.0 75 1.0

18 19 1.0 49 1.5

19

20 21 1.5 22 1.0 23 1.5

21 25 1.0

22 30 1.0

23 24 1.5 28 1.0

24 25 1.0 27 1.5

25

26 27 2.0 28 1.0 54 1.5

27 88 1.0

28

29 44 1.0 104 1.0 106 1.0

30 31 1.0 45 1.0 105 1.0

31

32 33 1.0 42 1.0 43 1.0

33 34 2.0 35 1.0

34

35 36 1.0

36 37 1.0 38 1.0 39 1.0

37

38

39

40

41

42

43

44

45

46 47 1.5 50 1.0 78 1.5

47 48 1.5 61 1.0

48 49 1.5 60 1.5

49 50 1.0

50

51 52 1.5 55 1.0 79 1.5

52 53 1.5 58 1.0

53 54 1.5 57 1.5

54 55 1.0

55

56 57 2.0 58 1.0 64 1.0

57 89 1.0

58

59 60 2.0 61 1.0 63 1.0

60 62 1.0

61

62

63 67 1.5 76 2.0

64 68 1.5 77 2.0

65 68 1.0 72 1.0 73 1.0 74 1.0

66 67 1.0 69 1.0 70 1.0 71 1.0

67

68

69

70

71

72

73

74

75

76

77

78 81 2.0 84 1.0

79 83 2.0 85 1.0

80 81 1.0 86 2.0 91 1.0

81 92 1.0

82 83 1.0 87 2.0 90 1.0

83 93 1.0

84

85

86

87

88

89

90

91

92 95 2.0 96 1.0

93 94 2.0 97 1.0

94

95

96 101 1.0 102 1.0 103 1.0

97 98 1.0 99 1.0 100 1.0

98

99

100

101

102

103

104

105 107 1.0 108 1.0 109 1.0

106 110 1.0 111 1.0 112 1.0

107

108

109

110

111

112

BD4

C	0.72712900	2.60935100	-0.15823600
C	-0.69443300	2.60704200	-0.16389000
C	-1.40639100	3.82266300	-0.31943000
C	-0.68556600	5.04426600	-0.33747300
C	0.71398900	5.05342200	-0.17463500
C	1.43776200	3.83570300	-0.09752300
N	1.12737000	6.32412900	-0.16959200
N	-1.10781300	6.30831300	-0.42717000

N	0.00438200	7.00297800	-0.31434100
C	-0.00582700	8.45311700	-0.42863300
C	2.80150500	3.49979800	-0.06103900
C	2.89622000	2.11215800	-0.13362900
N	1.63831300	1.56084000	-0.20455700
C	5.13048800	2.73210900	-0.09200600
C	4.22713100	1.66418700	-0.16044900
S	4.34373800	4.29121100	-0.00349100
C	6.46920700	2.33515200	-0.11036200
C	6.61277400	0.95980500	-0.19074400
S	5.05084300	0.15289500	-0.22649100
C	-2.86231400	2.10596400	-0.15571500
C	-2.77030400	3.48399000	-0.33434200
N	-1.60436900	1.56254300	-0.04001300
C	-4.19342100	1.66083100	-0.08786000
C	-5.09543500	2.71796300	-0.24478000
S	-4.31228400	4.26686200	-0.45272400
C	-6.58057100	0.95807800	-0.04337500
C	-6.43782500	2.32227000	-0.21884200
S	-5.01916500	0.15682700	0.07702200
C	1.41616500	0.23107300	-0.74551500
H	1.39344300	-0.53268000	0.03392600

C	-1.38387800	0.28286300	0.61096100
H	-1.35768500	-0.54356100	-0.10166900
H	-2.18789100	0.10454500	1.32573600
C	1.05035800	9.08831200	0.45194300
C	0.69633500	8.96513100	1.92181100
O	-0.38203500	8.61132000	2.30158600
O	1.63565300	9.30083300	2.82266400
C	2.94374100	9.68460400	2.42142500
H	3.45554900	9.97255300	3.33639300
H	3.48114700	8.85224400	1.96133800
H	2.92886000	10.53932900	1.74140200
H	0.16475200	8.71646900	-1.47520100
H	-1.00479400	8.76645000	-0.13553300
H	2.02407500	8.64408500	0.24421600
H	1.12467100	10.15107300	0.20011300
H	0.48015800	0.21263300	-1.29894000
H	2.21681000	-0.00631300	-1.44670800
H	-0.45052000	0.31120100	1.16860600
C	10.27634600	-0.48793100	-0.03235100
C	9.41454800	-1.50356200	-0.47356400
C	8.05815800	-1.11046900	-0.60466400
C	7.82982400	0.22103900	-0.22320400

S	9.30987900	0.95511200	0.25760500
C	-9.49526300	-1.63803900	0.53084000
C	-10.03566800	-0.48528900	-0.06042200
C	-9.09061900	0.54913800	-0.30195600
C	-7.78475500	0.20088600	0.07742300
S	-7.77334300	-1.36963700	0.77142400
C	-11.04123400	1.58794800	-0.93633400
C	-9.69338000	1.71976900	-0.83727800
S	-11.63501000	0.01810100	-0.45139600
C	7.96414900	-3.23693700	-1.50683600
C	7.25321200	-2.11833400	-1.20074900
S	9.65876900	-3.09816600	-1.06849100
H	6.20477000	-2.03903000	-1.44395400
C	7.32097300	-4.37446700	-2.22556300
C	-11.93555400	2.63809900	-1.45378400
C	-14.15791200	3.18384400	-1.94935600
C	8.75360300	-6.03626000	-1.16809600
O	7.84774000	-5.60376500	-2.18188400
O	-13.20881900	2.23627700	-1.45434500
H	8.64721000	-7.11791400	-1.13017900
H	9.78520700	-5.79383900	-1.42777700
H	8.50321300	-5.61588200	-0.19369900

H	-13.92865500	3.44371800	-2.98265300
H	-15.12358800	2.69123500	-1.88313600
H	-14.14311300	4.08772300	-1.34039300
H	7.31218600	3.01213000	-0.09006500
O	6.32193600	-4.18214800	-2.86311200
O	-11.55657100	3.71864000	-1.82306200
C	11.66345000	-0.32054800	0.08396400
C	-10.00140600	-2.81688200	1.09942400
C	12.54618700	-2.64477900	0.60996100
C	12.68745900	-1.24032100	0.19211200
C	14.10152100	-0.96885600	0.08492600
C	-11.71549600	-4.55538400	1.72933900
C	-12.10640400	-3.32320000	-0.23023500
C	-11.20277200	-3.47215200	0.92449700
H	11.96972200	0.72065100	0.07989400
H	-9.31756900	-3.27894900	1.80446500
C	13.94784700	-3.18023400	0.65606200
C	14.82756500	-2.21503300	0.36868700
C	-12.96259900	-5.02958800	1.11323500
C	-13.20105900	-4.32491000	0.00210600
C	-11.25552400	-5.12191500	2.89251600
C	14.78745000	0.17439900	-0.24338700

C	14.15419300	1.40924700	-0.54318400
C	16.20897300	0.18151800	-0.29794400
C	-10.06804600	-4.70652400	3.55058000
C	-11.96382000	-6.18763900	3.51405000
O	11.54296400	-3.23692700	0.92792800
O	-11.97167700	-2.62024400	-1.20115300
N	17.36229900	0.18535500	-0.34194800
N	13.64489900	2.41744400	-0.78329300
N	-9.10054400	-4.37741400	4.08836100
N	-12.53976000	-7.05205500	4.01730200
H	-7.27286900	3.00256300	-0.28027400
H	-9.19411600	2.62481200	-1.14643000
H	-13.57556000	-5.82595400	1.51306600
S	-14.53433600	-4.66807300	-1.08563500
O	-14.04919600	-4.78455100	-2.43253800
O	-15.37601400	-5.66574400	-0.47762100
Cl	-15.54807300	-2.84778200	-0.94594500
S	14.41562500	-4.79535100	1.15430700
O	13.70181700	-5.16904500	2.34320600
O	15.84495100	-4.91754800	1.03660400
Cl	13.58105200	-5.87830700	-0.43164800
H	15.90437300	-2.31290800	0.34220000

1 2 1.5 6 1.5 13 1.0

2 3 1.5 22 1.0

3 4 1.5 21 1.5

4 5 1.5 8 1.5

5 6 1.5 7 1.5

6 11 1.5

7 9 1.0

8 9 1.0

9 10 1.0

10 34 1.0 42 1.0 43 1.0

11 12 1.5 16 1.0

12 13 1.5 15 1.5

13 29 1.0

14 15 1.5 16 1.0 17 1.5

15 19 1.0

16

17 18 2.0 78 1.0

18 19 1.0 52 1.5

19

20 21 1.5 22 1.5 23 1.5

21 25 1.0

22 31 1.0

23 24 1.5 28 1.0

24 25 1.0 27 1.5

25

26 27 2.0 28 1.0 57 1.5

27 107 1.0

28

29 30 1.0 46 1.0 47 1.0

30

31 32 1.0 33 1.0 48 1.0

32

33

34 35 1.0 44 1.0 45 1.0

35 36 2.0 37 1.5

36

37 38 1.0

38 39 1.0 40 1.0 41 1.0

39

40

41

42

43

44

45

46

47

48

49 50 1.5 53 1.0 81 1.5

50 51 1.5 64 1.0

51 52 1.5 63 1.5

52 53 1.0

53

54 55 1.5 58 1.0 82 1.5

55 56 1.5 61 1.0

56 57 1.5 60 1.5

57 58 1.0

58

59 60 2.0 61 1.0 67 1.0

60 108 1.0

61

62 63 2.0 64 1.0 66 1.0

63 65 1.0

64

65

66 70 1.5 79 2.0

67 71 1.5 80 2.0

68 71 1.0 75 1.0 76 1.0 77 1.0

69 70 1.0 72 1.0 73 1.0 74 1.0

70

71

72

73

74

75

76

77

78

79

80

81 84 2.0 89 1.0

82 88 2.0 90 1.0

83 84 1.0 91 1.0 101 2.0

84 85 1.5

85 92 1.0 96 2.0

86 88 1.5 93 1.0 95 2.0

87 88 1.0 94 1.0 102 2.0

88

89

90

91 92 2.0 114 1.0

92 118 1.0

93 94 2.0 109 1.0

94 110 1.0

95 99 1.5 100 1.5

96 97 1.5 98 1.5

97 104 3.0

98 103 3.0

99 105 3.0

100 106 3.0

101

102

103

104

105

106

107

108

109

110 111 2.0 112 2.0 113 1.0

111

112

113

114 115 2.0 116 2.0 117 1.0

115

116

117

118

BD5

C	0.62202800	2.11145700	0.04105100

C	-0.80315300	2.13644200	-0.03213900
C	-1.47366800	3.36930100	-0.22804000
C	-0.73378500	4.57730200	-0.20494700
C	0.65361000	4.55904700	0.03345600
C	1.34550300	3.32730700	0.13849400
N	1.09189500	5.82182200	0.06209600
N	-1.12698700	5.85008800	-0.31621800
N	-0.00917800	6.52276700	-0.14203900
C	0.01586300	7.97188700	-0.26196400
C	2.70470200	2.97853000	0.21743400
C	2.79180000	1.59396600	0.13166700
N	1.52741700	1.05163700	0.02620600
C	5.03124600	2.21003000	0.18391800
C	4.12825900	1.14641900	0.09681200
S	4.24636500	3.76683900	0.29765000
C	6.37529800	1.81917600	0.14566700
C	6.52335900	0.45114800	0.02662400
S	4.96451300	-0.35808800	-0.01981100
C	-2.98563300	1.69891000	-0.17495600
C	-2.84398800	3.06979700	-0.34026300
N	-1.75284100	1.10992300	0.01974000
C	-4.33445600	1.29236300	-0.22069600
C	-5.18914500	2.37215200	-0.44329100
S	-4.34785300	3.89766600	-0.58040600

C	-6.74087500	0.66185100	-0.36233700
C	-6.54579800	2.01727400	-0.52024500
S	-5.21872400	-0.18608100	-0.12807800
C	1.32839900	-0.25838800	-0.58928100
C	-1.65976100	-0.10540700	0.83215900
H	-2.63525600	-0.59035700	0.76679700
C	1.07155400	8.58297800	0.63585700
C	0.67823500	8.49516600	2.09835100
O	-0.43500200	8.24158900	2.45699200
O	1.62344300	8.75403600	3.01922300
C	2.97227700	9.00097200	2.64678100
H	3.48792700	9.24937400	3.57128600
H	3.43461600	8.11292600	2.21001300
H	3.05877600	9.84362000	1.95693400
H	0.21214500	8.22763400	-1.30605000
H	-0.97981200	8.31222700	0.01194900
H	2.03332100	8.10428000	0.45389400
H	1.18534500	9.64061800	0.37624700
H	2.00702300	-0.33261900	-1.44573600
H	-0.95000400	-0.79954900	0.38880900
C	10.18654700	-1.01909600	0.15776500
C	9.33234700	-2.00194400	-0.34427600
C	7.97464000	-1.59669700	-0.47767500
C	7.74354400	-0.29094000	-0.03279300

S	9.22086900	0.40937300	0.50201600
C	-9.80311100	-1.80844500	0.02096700
C	-10.21373900	-0.70315100	-0.72669400
C	-9.20235300	0.28309800	-0.92462700
C	-7.97820200	-0.06151000	-0.34553200
S	-8.12297000	-1.56350700	0.47199800
C	-11.00262600	1.30746500	-1.92128400
C	-9.67721900	1.41260000	-1.64581700
S	-11.72551100	-0.19497200	-1.38661600
C	7.88761900	-3.68156000	-1.47208700
C	7.17494500	-2.57573100	-1.12849100
S	9.58020700	-3.57461300	-1.00476700
H	6.12975600	-2.47956400	-1.38030500
C	7.25657000	-4.78399600	-2.24727600
C	-11.76901800	2.32937400	-2.65086600
C	-13.87195500	2.89834200	-3.50502800
C	8.60094800	-6.49553200	-1.15959800
O	7.77011700	-6.02357800	-2.21759100
O	-13.04851000	1.96797900	-2.80218000
H	8.46363900	-7.57475900	-1.14427800
H	9.65214500	-6.27538500	-1.35119400
H	8.30542100	-6.08031500	-0.19561400
H	-13.48967700	3.05796200	-4.51339200
H	-14.86023200	2.44833400	-3.53717300

H	-13.90082300	3.85331700	-2.97986500
H	7.21508500	2.49947900	0.17740700
O	6.28524400	-4.56866200	-2.91997300
O	-11.30325300	3.36298900	-3.05629200
C	11.58062000	-0.84851300	0.30924300
C	-10.41507000	-2.93597100	0.61724600
C	12.53001500	-3.14859900	0.66521300
C	12.63549700	-1.72085700	0.39682700
C	-12.40010500	-3.49117400	-0.82159300
C	-11.58673900	-3.60707100	0.38468300
H	11.89903500	0.19187300	0.34506400
H	-9.85859000	-3.35047500	1.45614300
O	11.48492500	-3.72684900	0.90085300
O	-12.07593200	-2.84778300	-1.80093000
H	-7.35600400	2.72066800	-0.63815600
H	-9.09739800	2.26668600	-1.96072600
H	-13.32890400	-4.08211300	-0.82176600
H	13.48715200	-3.69140300	0.70053900
C	13.97175500	-1.07326700	0.37978500
C	-11.91506500	-4.64412100	1.39476700
O	-11.23529500	-4.90512100	2.35882200
O	14.15928000	0.11757000	0.30934400
H	0.32176100	-0.28061700	-1.00604600
C	-1.31238100	0.15813400	2.29425900

C	1.56671000	-1.43109400	0.35460300
H	-1.26039500	-0.81600600	2.79243000
H	-0.30788800	0.58791500	2.35338500
H	2.58420500	-1.37851300	0.75089900
H	0.90885700	-1.34473400	1.22290600
O	-13.07331900	-5.26679100	1.12211900
O	14.97343300	-1.96373600	0.44815800
C	-13.48980200	-6.28690400	2.04435000
C	-12.88544600	-7.63195700	1.68599100
C	16.30184400	-1.41517200	0.43956400
C	17.28301000	-2.56441200	0.42823500
H	-13.21328800	-5.97803300	3.05223900
H	-14.57689000	-6.30999100	1.95514100
H	-13.08659400	-7.82519500	0.62804300
H	-11.79959500	-7.57162700	1.80374200
H	16.42367300	-0.78533000	1.32340000
H	16.41181000	-0.77579600	-0.43988200
H	17.10358400	-3.19408400	1.30622700
H	18.28625200	-2.14094800	0.55079500
C	-13.43661400	-8.78045700	2.53045300
C	-13.08237800	-8.69931600	4.01174700
C	17.23405900	-3.41034500	-0.83962100
C	18.23264400	-4.55950400	-0.82060900
H	-14.52567500	-8.82758800	2.41356000

H	-13.05143800	-9.71913400	2.12166200
H	-13.53196100	-7.82946900	4.49600200
H	-13.43241900	-9.58494800	4.54639600
H	-12.00054300	-8.63048400	4.15251500
H	16.22134300	-3.80172600	-0.97011200
H	17.42794800	-2.76740900	-1.70572100
H	19.25805600	-4.19296600	-0.72021900
H	18.04091300	-5.23523300	0.01731800
H	18.17998800	-5.14816800	-1.73878500
C	1.35823700	-2.76682600	-0.34624000
C	-2.30642800	1.06039300	3.01175200
H	-3.31634500	0.64323500	2.98412000
H	-2.02561100	1.18509300	4.05887300
H	-2.34252900	2.05431100	2.56115900
H	1.53868700	-3.59849100	0.33608100
H	0.33740600	-2.86609000	-0.72589300
H	2.03747000	-2.88266200	-1.19443000

1 2 1.5 6 1.5 13 1.0

2 3 1.5 22 1.0

3 4 1.5 21 1.5

4 5 1.5 8 1.5

5 6 1.5 7 1.5

6 11 1.5

7 9 1.0

8 9 1.0

9 10 1.0

10 32 1.0 40 1.0 41 1.0

11 12 1.5 16 1.0

12 13 1.5 15 1.5

13 29 1.0

14 15 1.5 16 1.0 17 1.5

15 19 1.0

16

17 18 2.0 75 1.0

18 19 1.0 49 1.5

19

20 21 1.5 22 1.5 23 1.5

21 25 1.0

22 30 1.0

23 24 1.5 28 1.0

24 25 1.0 27 1.5

25

26 27 2.0 28 1.0 54 1.5

27 88 1.0

28

29 44 1.0 96 1.0 98 1.0

30 31 1.0 45 1.0 97 1.0

31

32 33 1.0 42 1.0 43 1.0

33 34 2.0 35 1.0

34

35 36 1.0

36 37 1.0 38 1.0 39 1.0

37

38

39

40

41

42

43

44

45

46 47 1.5 50 1.0 78 1.5

47 48 1.5 61 1.0

48 49 1.5 60 1.5

49 50 1.0

50

51 52 1.5 55 1.0 79 1.5

52 53 1.5 58 1.0

53 54 1.5 57 1.5

54 55 1.0

55

56 57 2.0 58 1.0 64 1.0

57 89 1.0

58

59 60 2.0 61 1.0 63 1.0

60 62 1.0

61

62

63 67 1.5 76 2.0

64 68 1.5 77 2.0

65 68 1.0 72 1.0 73 1.0 74 1.0

66 67 1.0 69 1.0 70 1.0 71 1.0

67

68

69

70

71

72

73

74

75

76

77

78 81 2.0 84 1.0

79 83 2.0 85 1.0

80 81 1.0 86 2.0 91 1.0

81 92 1.0

82 83 1.0 87 2.0 90 1.0

83 93 1.0

84

85

86

87

88

89

90

91

92 95 2.0 104 1.5

93 94 2.0 103 1.5

94

95

96

97 99 1.0 100 1.0 132 1.0

98 101 1.0 102 1.0 131 1.0

99

100

101

102

103 105 1.0

104 107 1.0

105 106 1.0 109 1.0 110 1.0

106 111 1.0 112 1.0 117 1.0

107 108 1.0 113 1.0 114 1.0

108 115 1.0 116 1.0 119 1.0

109

110

111

112

113

114

115

116

117 118 1.0 121 1.0 122 1.0

118 123 1.0 124 1.0 125 1.0

119 120 1.0 126 1.0 127 1.0

120 128 1.0 129 1.0 130 1.0

121

122

123

124

125

126

127

128

129

130

131 136 1.0 137 1.0 138 1.0

132 133 1.0 134 1.0 135 1.0

133

134

135

136

137

138
